# The crucial role of mitochondrial/chloroplast-related genes in viral genome replication and host defense: integrative systems biology analysis in plant-virus interaction

**DOI:** 10.3389/fmicb.2025.1551123

**Published:** 2025-04-23

**Authors:** Amir Ghaffar Shahriari, Aminallah Tahmasebi, Mohamad Hamed Ghodoum Parizipour, Zahra Soltani, Ahmad Tahmasebi, Muhammad Shafiq Shahid

**Affiliations:** ^1^Department of Agriculture and Natural Resources, Higher Education Center of Eghlid, Eghlid, Iran; ^2^Department of Agriculture, Minab Higher Education Center, University of Hormozgan, Bandar Abbas, Iran; ^3^Department of Plant Protection, Faculty of Agriculture, Agricultural Sciences and Natural Resources University of Khuzestan, Mollasani, Iran; ^4^Institute of Biotechnology, School of Agriculture, Shiraz University, Shiraz, Iran; ^5^Department of Plant Sciences, College of Agricultural and Marine Sciences, Sultan Qaboos University, Muscat, Oman

**Keywords:** viral infections, biomarker identification, differentially expressed genes, meta-analysis, plant organelle responses

## Abstract

Plant viruses participate as biotrophic parasites in complex interactions with their hosts, resulting in the regulation of a diverse range of chloroplast/mitochondria-related genes that are essential for mediating immune responses. In this study, integrative systems biology approaches were applied to identify chloroplast/mitochondrial genes during viral infections caused by a wide number of viruses in *Arabidopsis thaliana*, tobacco (*Nicotiana tabacum L.*), and rice (*Oryza sativa L.*). These findings indicated that 1.5% of the DEGs were common between *Arabidopsis*/tobacco and *Arabidopsis*/rice, whereas 0.1% of the DEGs were shared among all species. Approximately 90% of common DEGs are uniquely associated with chloroplasts and mitochondria in the host defense against viral infection and replication. The functions of WRKY, NAC, and MYB transcription factors in imparting resistance to viral infections can be established. Promoter analysis revealed that AP2/EREBP, DOF, and C2H2 zinc finger factors included the most frequent binding sites and played a more important role in plant-viral interactions. Comparative analysis revealed several miRNAs with defensive functions including miRNA156, miRNA160, and miRNA169. The PPI network revealed several key hub genes mostly related to chloroplasts/mitochondria, including *ZAT6*, *CML37*, *CHLI*, *DREB*, *F27B13*.20, and *ASP2* with upregulation, also *PLGG1*, *PSBY*, *APO2*, *POR*, *ERF*, and *CSP* with downregulation. Moreover, novel hub genes with unknown functions, such as *AT2G41640* and *AT3G57380* have been identified. This study represents the first preliminary systems biology approach to elucidate the roles of chloroplast/mitochondria-related genes in *Arabidopsis*, tobacco, and rice against viral challenges by introducing valuable candidate genes for enhanced genetic engineering programs to develop virus-resistant crop varieties.

## 1 Introduction

Plant viruses are an economically important group of pathogens that significantly reduce crop yield in any global cultivation system. These entities are mainly composed of two macromolecules, proteins and nucleic acids, which act as coats and genomes, respectively (Xavier and Whitfield, 2023). Plant-infecting viruses, as obligate biotrophic pathogens, depend significantly on host molecular systems for genome replication (Heinlein, 2015). Viral infections are mostly associated with several symptoms that adversely affect the morphology and physiology of virus-infected host plants (Takahashi et al., 2019). This phenomenon eventually results in impaired plant integrity by reducing biomass and/or crop yield (Paudel and Sanfaçon, 2018). Viruses have developed sophisticated mechanisms to manipulate and reprogram the metabolism of host cells, which not only aids in their replication but also enhances pathogenesis dynamics while inhibiting plant immune systems. These pathogens can effectively take over host components, such as proteins, phospholipids, intracellular membranes, and bioactive compounds, resulting in the formation of viral replication complexes (VRCs), which serve as specialized sites for viral assembly and maturation. Such VRCs can employ diverse organelles, including the endoplasmic reticulum, mitochondria, vacuoles, Golgi apparatus, peroxisomes, tonoplasts, chloroplasts, and plasma membrane to facilitate their lifecycle (Budziszewska and Obrepalska-Steplowska, 2018; Bwalya and Kim, 2023).

Chloroplasts are plant membrane-bound organelles that are usually affected during viral infection, leading to altered chlorophyll content and reduced photosynthesis in the infected plant (Bhattacharyya and Chakraborty, 2018). This effect may be recognized by the development of leaf chlorosis, which is a frequent viral symptom (He et al., 2021). Therefore, chloroplasts facilitate photosynthesis and contribute significantly to antiviral defense by producing JA/SA hormones, reactive oxygen species (ROS) generation, and interorganellar signaling (Zanardo et al., 2019; Zhao et al., 2016). It is generally accepted that plant viruses modify chloroplasts to utilize their physical structures and biochemical components for replication (Bhattacharyya and Chakraborty, 2018). Mitochondria are other plant cell organelles that plays a role in plant-virus interactions (Wang et al., 2022). The main biological importance of mitochondria is in energy cycling and carbohydrate metabolism within the cells (Liberatore et al., 2016). This organelle has also been reported to be involved in plant defense against viral infections (Blake et al., 2007; Ordog et al., 2002; Song et al., 2009). Mitochondria are mainly responsible for antiviral defense by mediating immune signaling and are subsequently involved in programmed cell death (Wang et al., 2022). Furthermore, chloroplasts/mitochondria may be involved in plant defense against viral invasion by triggering an immune response in virus-challenged cells (Ershova et al., 2022). Several chloroplast and mitochondrial proteins have been identified that interact with viral proteins to facilitate viral pathogenicity and/or plant antiviral defenses (Bhattacharyya and Chakraborty, 2018).

Most plant viruses, including the *Plum pox virus* (PPV), *Turnip mosaic virus* (TuMV), and *Tobacco mosaic virus* (TMV), interact with chloroplast VRCs for replication, leading to adverse morphological effects on organelles (Bwalya and Kim, 2023). Similarly, other RNA viruses, including the *Cucumber mosaic virus* (CMV), cause significant economic losses to cropping systems annually and severely damage the structures of chloroplasts and mitochondria upon infecting susceptible plant cultivars (Shen et al., 2024). It has been shown that replicas of TMV interact with the ATP synthase of chloroplasts, regulating the antiviral defense response in virus-infected plants (Bwalya and Kim, 2023). The disease-specific protein of the *Rice stripe virus* (RSV) interacts with the chloroplast PsbP protein, leading to disruptions in chloroplast function and resulting in the accumulation of RSV (Kong et al., 2014). Successful infection by DNA viruses, such as *Cabbage leaf curl virus* (CaLCuV) and *Cauliflower mosaic virus* (CaMV), is linked to the over-expression of chloroplast-related genes. These interactions disrupt normal chloroplast function, facilitate viral replication, and exacerbate diseases in susceptible plant cultivars (Angel et al., 2013). Although numerous studies have been carried out to reveal the role of chloroplast/mitochondria in viral infection, the specific mechanisms by which few viruses, such as *Spinach curly top virus* (SCTV), *Hibiscus latent Singapore virus* (HLSV), *Potato virus A* (PVA), and *Rice tungro spherical virus* (RTSV), interact with these plant organelles remain largely unknown.

To develop effective molecular approaches for improving plant resistance, it is crucial to adopt a comprehensive perspective on the complex mechanisms that regulate plant–viral interactions, as well as to identify all functional components associated with viruses that contribute to infection. Although extensive research has been conducted on the role of chloroplast/mitochondria in plant-virus interactions, the genetic basis of this interaction has been poorly studied. Transcriptomics has proven to be a useful approach for explore the potential genes associated with these interactions in model and cultivated plants. This includes a series of multi-omics systems biology approaches applied to determine the gene expression of host plants in response to viral infection (Gilbert and Hughes, 2011).

This study was conducted to identify the expression patterns of chloroplast/mitochondrion-related genes involved in host responses to various viral infections in *Arabidopsis*, tobacco, and rice plants. Furthermore, the gene network was depicted, genes differentially expressed in virus-infected plants, and hub genes involved in the interactions were determined. In this study, we used transcriptome data to identify key genes and modules associated with chloroplast/mitochondrial responses in plant-virus interactions. We also highlight the important TFs, biological processes, and pathways that can be considered potential chloroplast/mitochondrion-based biomarkers and targets in plant-virus interactions.

## 2 Methods

**Figure 1:**
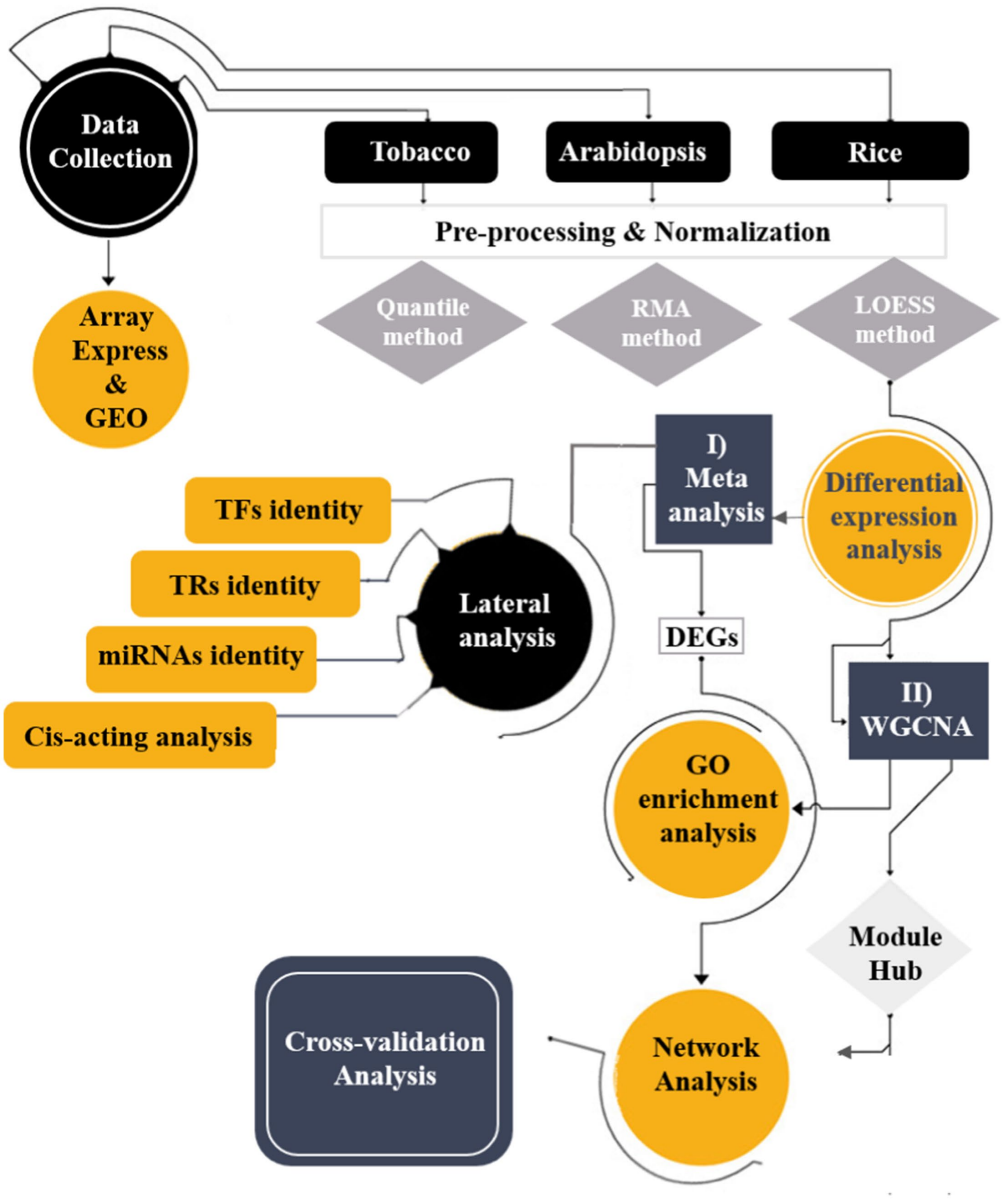
Schematic overview of applied integrative systems biology approaches to understand the transcriptome responses of plant species to viral infections.

The overall framework integrated the transcriptome datasets of three plant species (*Arabidopsis* [*Arabidopsiss thaliana*], tobacco [*Nicotiana tabacum*], and rice [*Oryza sativa*]) through systems biology approaches to detect crucial mitochondrial/chloroplast-related genes against viral infections (Figure 1). First, the meta-analysis identified differentially expressed genes (DEGs), followed by co-expression gene network analysis, which grouped DEGs into distinct modules.

### 2.1 Data collecting

Raw microarray datasets of three plant species, tobacco, *Arabidopsis*, and rice exposed to viral infections, were retrieved from the Array Express^1^ and Gene Expression Omnibus (GEO)^2^ databases (Table 1). The literature provides insights into the typical symptoms observed in plant-virus interactions involving mosaic symptoms, stripe symptoms, leaf malformation, chlorosis, necrosis, stunting, leaf curling, and reduced tillering. These symptoms are often associated with structural and functional changes in the chloroplasts and mitochondria of the infected plants, such as swelling, starch accumulation, grana disintegration, and reduced photosynthetic efficiency. Data selection was performed based on Minimal Information about Microarray Experiment (MIAME) requirements (Brazma et al., 2001). Thus, experiments with biological and technical replicates of treatments and non-treatments, excluding mutated or transgenic samples with relatively similar genetic backgrounds, were selected (Cohen and Leach, 2019; Soltani et al., 2023). A total of 13 studies, including 32 tobacco samples, 91 *Arabidopsis* samples, and 28 rice samples, were selected for comparative analysis (Table 1). The collected datasets consisted of two platforms, Affymetrix (Accession: GPL198 and GPL2025) and Agilent (Accessions: GPL10098 and GPL892). Probe-gene maps and annotation files were obtained from the Affymetrix database.

**Table 1:** Samples retrieved from GEO and Array Express for comparative analysis.

Accession	Acronym	Virus name	Platform	Control	Treatment	Organism
E-GEOD-47180	HLSV	*Hibiscus latent Singapore virus*	GPL10098, Agilent	3	9	*Nicotiana tabacum*
	TMV	*Tobacco mosaic virus*				
E-GEOD-47410	CMV	*Cucumber mosaic virus*	GPL10098, Agilent	3	9	*Nicotiana tabacum*
E-GEOD-42833	PVA	*Potato virus A*	GPL10098, Agilent	2	6	*Nicotiana tabacum*
E-GEOD-20278	TuMV	*Turnip mosaic virus*	GPL198, Affymetrix	6	6	*Arabidopsis thaliana*
E-GEOD-37921	CMV	*Cucumber mosaic virus*	GPL198, Affymetrix	3	3	*Arabidopsis thaliana*
E-GEOD-62180	CaLCuV	*Cabbage leaf curl virus*	GPL198, Affymetrix	15	30	*Arabidopsis thaliana*
	SCTV	*Spinach curly top virus*				
E-GEOD-11217	PPV	*Plum pox virus*	GPL198, Affymetrix	12	12	*Arabidopsis thaliana*
E-GEOD-9408	CaMV	*Cauliflower mosaic virus*	GPL198, Affymetrix	2	2	*Arabidopsis thaliana*
E-GEOD-11025	RSV	*Rice stripe virus*	GPL2025, Affymetrix	6	6	*Oryza sativa*
E-GEOD-6126	RSV	*Rice stripe virus*	GPL892, Agilent	2	2	*Oryza sativa*
E-GEOD-6125	RTSV	*Rice tungro spherical virus*	GPL892, Agilent	2	2	*Oryza sativa*
E-GEOD-6124	RTSV	*Rice tungro spherical virus*	GPL892, Agilent	2	2	*Oryza sativa*
E-GEOD-14606	RTSV	*Rice tungro spherical virus*	GPL892, Agilent	2	2	*Oryza sativa*

### 2.2 Pre-processing and normalization

Pre-processing and background correction of the Affymetrix raw data were performed using Expression Console software with a robust multi-chip average (RMA) approach. After background correction, quantile normalization was performed, and the Median Polish method was applied to summarize probe sets (Gautier et al., 2004; Irizarry et al., 2003). In addition, Agilent raw data (two colors) were processed in two steps through Flex Array software (Ver 1.6.3). Background correction was applied using the NormExp algorithm and the Maximum Likelihood Estimation (MLE) method. Normalization between arrays was then applied using the quantile approach. Agilent raw data (one color) were normalized in the R program using the LIMMA and LOESS packages (Bolstad et al., 2003). We applied the SVA package and empirical Bayes approach to the R software to correct the batch effect (Johnson et al., 2007; Leek et al., 2012). Finally, gene sequences were retrieved from the NCBI/Batch Entrez database. Nucleotide sequences of each gene were BLASTed against TAIR using CLC bio software, and the probeset IDs were converted into *Arabidopsis thaliana* orthologs (Soltani et al., 2023).

### 2.3 Meta-analysis and identification of DEGs

First, control and treatment samples in each study were defined separately. Meta-analysis was performed individually on the tobacco, *Arabidopsis*, and rice datasets using the MetaDE R package. This package is based on the rank Prod method to rank genes based on fold change (FC) and find robust DEGs (Ma et al., 2019). A comparative analysis was conducted between the two classes for each of the three species, utilizing a moderated t-statistic with 1,000 random permutations to describe DEGs with significant expression. Converting FC into ranks overcomes heterogeneity among multiple datasets, and an overall ranked gene list is produced based on the False Discovery Rate (*FDR*) of each gene. In this approach, upregulated and downregulated genes with log2 FC > 1 or log2 FC < −1 and *FDR* ≤ 0.05 were identified as significant DEGs (Balan et al., 2017).

### 2.4 Gene co-expression network analysis

Weighted gene co-expression network analysis (WGCNA) in R space was performed on the normalized expression values of DEGs (*p-*value ≤ 0.05) to identify gene groups with similar expression patterns (Langfelder and Horvath, 2008). First, a similarity matrix was calculated based on the Pearson correlation coefficient for each DEG pair. The similarity matrix was converted to adjacency matrices by raising them to power (*β*), which makes the adjacency matrix a continuous value between 0 and 1, and the co-expression similarity was raised to achieve a scale-free topology. Next, adjacency was transformed into a topological overlap matrix (TOM), and DEGs were hierarchically clustered based on TOM similarity. To detect highly correlated modules, a dissimilarity matrix was obtained (dissTOM), which was applied to represent the distances between the DEGs (Montenegro, 2022; Soltani et al., 2023). Finally, a dynamic tree cut algorithm was used to discover the co-expression gene modules. The eigengene module was used to summarize the expression patterns of each module. The parameters used for each dataset included *Arabidopsis* datasets (height cut of 0.6 and minimum module size of 30 genes), tobacco datasets (height cut of 0.9 and minimum module size of 100 genes), and rice datasets (height cut of 0.3 and minimum module size of 30 genes) to avoid abnormal modules in the dendrogram.

### 2.5 Identification of hub genes

Calculation of genes with Pearson’s coefficient correlation above the cut-off value, i.e., ±0.3 were used for making of biological network by Cytoscape software version 3.6.1 (Shannon et al., 2003). To discover hub genes, computational algorithms of Maximal Clique Centrality (MCC) were used through a plug-in of Cytoscape, CytoHubba (Chin et al., 2014; Li et al., 2020) and visualizing the 30 nodes with the highest interaction in the biological network.

### 2.6 Functional enrichment analysis

To determine the biological significance of the DEGs or modules identified through systems biology analysis, we performed Gene Ontology (GO) and Kyoto Encyclopedia of Genes and Genomes (KEGG) enrichment analysis. The Enrichment analysis included, Biological Process (BP), Molecular Function (MF), and Cellular Component (CC) categories, which were conducted using the Database for Annotation, Visualization, and Integrated Discovery (DAVID) web server (Shahriari et al., 2022) and g:Profiler (Raudvere et al., 2019). GO terms with a corrected *p-*value ≤ 0.05 were classified as significant (Shaar-Moshe et al., 2015).

### 2.7 Identification of TFs, TRs, and miRNAs families

The sequences of DEGs from all three datasets were acquired through a BLASTx search and subsequently analyzed against the iTAK database using default parameters^3^ to identify transcription factors (TFs) and transcriptional regulators (TRs) (Zheng et al., 2016). Next, the psRNATarget database (Dai et al., 2018) and standard settings of hpsize of 20, flanking length of 17 bp upstream and 13 bp downstream, and translation inhibition range of 9–11 nucleotides were applied to discover candidate miRNAs. Finally, the genes targeted by the miRNAs were evaluated using GO analysis.

### 2.8 Cis-acting element analysis

To discover conserved cis-acting elements, 1,500 bp upstream flanking regions of DEGs were downloaded from the Ensemble database.^4^ All conserved motifs were retrieved from the MEME website^5^ with an *E-value* threshold of < 10e − 4 (Bailey et al., 2009). Thus, we used the Tomtom v 5.0.1 tool^6^ to eliminate redundant motifs and detect known CREs based on the motif database of JASPAR CORE 2024 plants (Gupta et al., 2007; Khan et al., 2018), with a threshold *E-value* cutoff of 0.05, and sequences with significant similarity to known motifs were retained. The GOMO tool (Buske et al., 2010) was used to determine the biological roles of cis-acting elements.

### 2.9 Protein–protein interactions networks

Protein–protein interactions (PPI) network analysis was performed based on the protein information extracted from the three datasets to identify plausible interactions among proteins with candidate hub genes. The common list of TAIR IDs for each of the three species was BLASTed against the STRING database^7^ (Szklarczyk et al., 2015) with default parameters (required interaction score = 0.15 and 0.4). Finally, the networks were constructed using Cytoscape software, and crucial hub genes in the networks were screened out by node degree.

### 2.10 Cross-validation analysis

In order to validate of results, a leave-one-out cross-validation (LOOCV) was performed on the expression values of hub genes in all three species obtained through co-expression analysis (Shahriari et al., 2022; Tahmasebi and Niazi, 2021). During cross-validation analysis, the initial dataset was split into training and test sets. Finally, one sample from the initial dataset was consecutively discarded for testing, and the others for training. We applied model evaluation indexes, such as the area under the curve (AUC), which were calculated as a quantitative measure of the model quality.

## 3 Results

### 3.1 Pre-processing and normalization

Pre-processing and normalization were conducted using two different approaches, RMA and LOESS, which identified noise and removed or reduced the effect of these noises on the machine-learning algorithm. The application of RMA successfully addressed systematic biases, ensuring that expression levels across various samples remained comparable. In contrast, LOESS fits a smooth curve to the data points, facilitating the adjustment of local variations and minimizing the influence of outliers. By identifying and mitigating noise, the reliability of the predictive models was improved, enabling them to capture the underlying patterns in the data better. This comprehensive approach to pre-processing and normalization ensures that the findings are robust and reproducible, ultimately contributing to the validity of the research outcomes.

### 3.2 Meta-analysis and identification of DEGs

The systems biology approach has been used in several transcriptome experiments, including *Arabidopsis*, tobacco, and rice plants, to identify specific gene expression changes associated with chloroplasts and mitochondria involved in host plant responses to viral infections. Thus, the adjusted t-test and Rank Prod approach were applied to analyze the microarray data and detect DEGs.

Based on these results, a meta-analysis of *Arabidopsis* datasets revealed 1,811 significant DEGs (*FDR* ≤ 0.05). Among the DEGs, 748 and 1,063 upregulated and downregulated genes were discovered, respectively (Supplementary Table S1). Meta-analysis of tobacco datasets, 1,749 upregulated and 1,949 downregulated genes were detected (Supplementary Table S1). In the case of the rice datasets, 232 upregulated and 372 downregulated genes were extracted (Supplementary Table S1).

**Figure 2:**
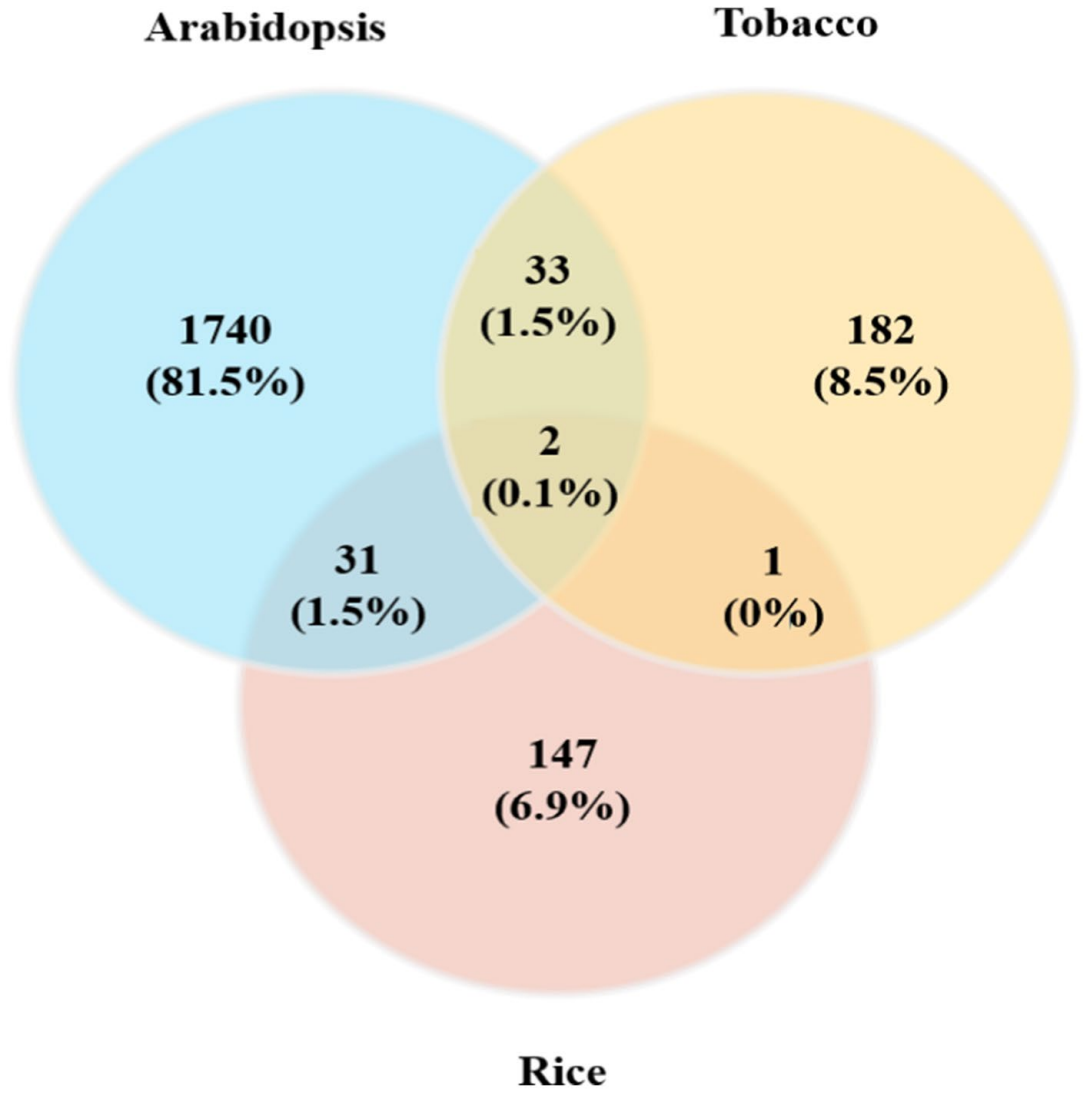
Comparison of DEGs obtained from individual meta-analyses of *Arabidopsis*, tobacco, and rice plants with different viral infections. A Venn diagram of common genes in the three plant species whose expression was upregulated/downregulated in response to viral infections is presented.

Moreover, we reported 33 (1.5%) common genes between the *Arabidopsis* and tobacco datasets, 31 (1.5%) common genes between the *Arabidopsis* and rice datasets, and finally one common gene between the tobacco and rice datasets (Figure 2; Supplementary Table S1). Only two (0.1%) DEGs were identified as being commonly expressed in all plant species (Figure 2). The identification of common genes among *Arabidopsis*, tobacco, and rice suggests that these species utilize similar genetic frameworks in response to viral infections. These common DEGs revealed critical insights into the evolutionary and functional adaptations that play crucial roles in plant defense mechanisms, overlapping signaling networks, and metabolic processes. Such comparative analyses may play pivotal roles in the regulation of mitochondrial and chloroplast functions during viral challenges, reflecting a conserved evolutionary strategy for pathogen resistance.

### 3.3 Functional enrichment analysis of DEGs

**Figure 3:**
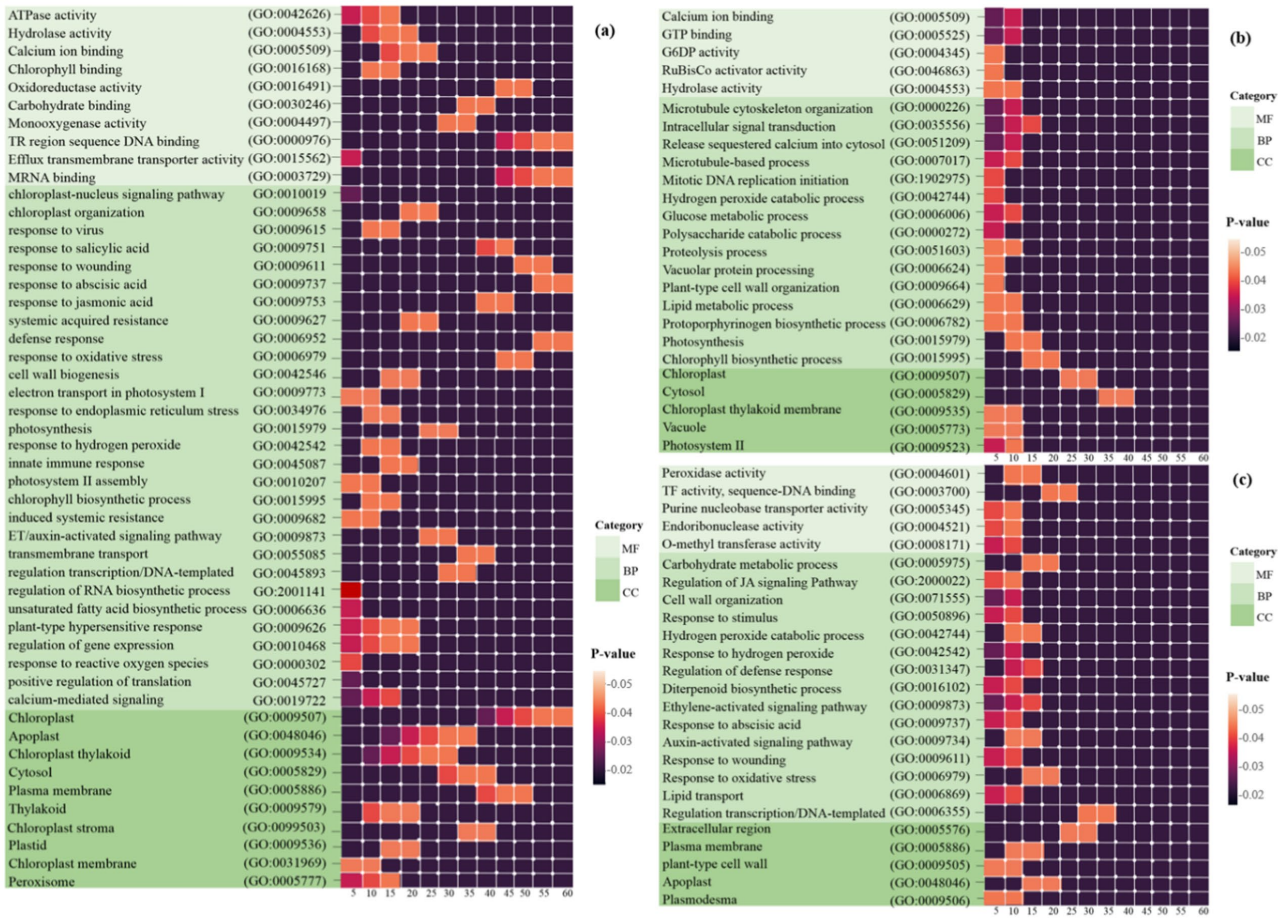
Heat map plots of the enrichment analysis for each DEG in **(a)** *Arabidopsis* **(b)** tobacco and **(c)** rice plants are presented using DAVID. The intensity of the colors indicates the degree of regulation of DEGs based on a *p*-value ≤ 0.05, which was used to identify the statistical significance of the GO pathway enrichment. The horizontal axis represents the number of genes associated with each pathway. MF, molecular function; BP, biological process; CC, cellular component.

GO analysis was performed to determine the functions of DEGs during plant-virus interactions. Heat map plots showing the expression of several DEGs associated with BP, CC, and MF were generated to assess functional similarities and differences in each of the three types of datasets (Figure 3). These findings suggest that mitochondria and chloroplasts display both common and distinct molecular responses essential for survival during viral infections, highlighting the importance of understanding these interactions.

Initially, 1,811 *Arabidopsis* DEGs were explored for GO analysis (Figure 3a). These genes were associated with the processes of response to virus (GO: 0009615), defense response (GO: 0006952), response to reactive oxygen species (GO: 0000302), induced systemic resistance (ISR) (GO: 0009682), response to oxidative stress (GO: 0006979), plant-type hypersensitive response (GO: 0009626), chloroplast-nucleus signaling pathway (GO: 0010019), innate immune response (GO: 0045087), response to SA/ABA/JA/ET hormones, and calcium-mediated signaling (GO: 0019722). In the CC category, the most significantly enriched GO terms were chloroplast, chloroplast thylakoid, cytosol, chloroplast stroma, plasma membrane and chloroplast membrane. In the MF category, chlorophyll binding, mRNA binding, oxidoreductase activity, ATPase activity, and protein binding were observed (Figure 3a).

A total of 3,698 tobacco DEGs were examined. Pathways such as the chlorophyll biosynthetic process (GO: 0015995), photosynthesis (GO: 0015979), proteolysis involved in cellular protein catabolic processes (GO: 0051603), and hydrogen peroxide catabolic processes (GO: 0042744) were strongly expressed in BP. In the MF category, calcium ion binding and GTP binding are most directly linked to the chloroplast/mitochondrial response in plant defense against viral infections, whereas other functions, including glucose-6-phosphate dehydrogenase activity, hydrolase activity, and ribulose-1,5-bisphosphate carboxylase, contribute indirectly through metabolic support and signaling pathways. In addition, the CC class included chloroplast, cytosol, chloroplast thylakoid membrane, and photosystem II (Figure 3b).

Finally, GO analysis of the 604 DEGs responsive to rice was performed. Processes such as response to oxidative stress (GO: 0006979), response to hydrogen peroxide (GO: 0042542), regulation of defense response (GO: 0031347), response to ABA (GO: 0009737), regulation of the JA-mediated signaling pathway (GO: 2000022), and auxin/ethylene-activated signaling pathway (GO: 0009873) were strongly expressed in BP. In the CC category, the most significantly enriched GO terms were plasma membrane, plant-type cell wall, apoplast, and plasmodesma. In the MF category, peroxidase, O-methyltransferase, transcription factor, purine nucleobase transmembrane transporter, and endoribonuclease activity was observed (Figure 3c).

Comparative analysis of DEGs revealed that while both mitochondria and chloroplasts are integral to the plant’s defense mechanisms against viral infections, a total of 576 and 36 chloroplast-related genes are more directly regulated in *Arabidopsis* and tobacco viral interactions, respectively, whereas mitochondrial genes mainly regulate energy metabolism and ROS responses and play a complementary role in maintaining plant survival during viral challenges.

GO enrichment analysis suggested that viral proteins can target chloroplasts and mitochondria to manipulate host defenses. Therefore, it is plausible that mitochondrial/chloroplast intricate functions not only facilitate viral replication but also enhance the plant’s defensive mechanisms, underscoring the necessity for further research into the roles of these organelles in plant-virus interactions.

### 3.4 Common and unique DEGs associated with viral resistance in mitochondrial/chloroplast

**Figure 4:**
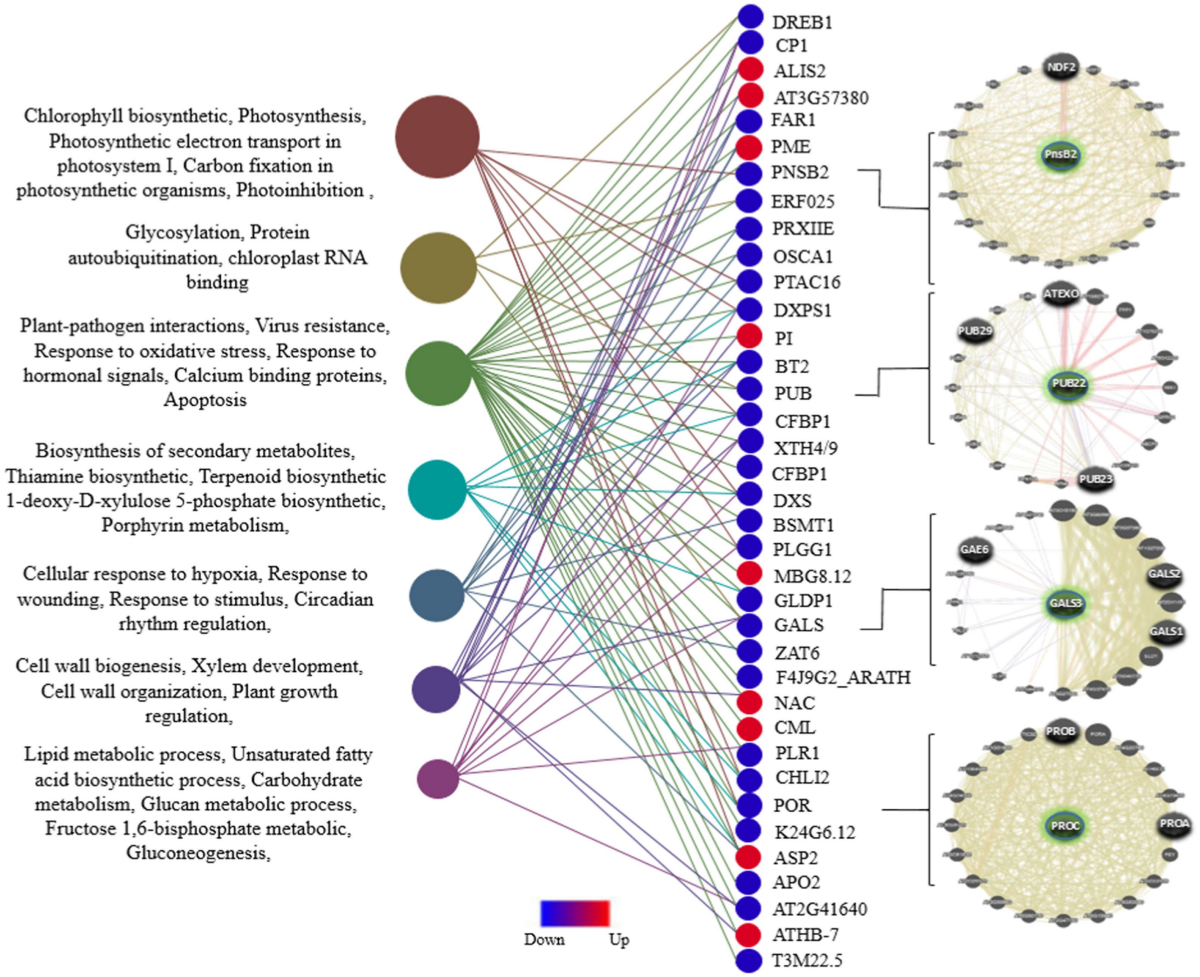
Distribution of GO enrichment for DEGs common across all three plant species, including *Arabidopsis*, tobacco, and rice. A total of 47 common and Unique DEGs (*p*-value ≤ 0.05) associated with viral resistance in the mitochondria and chloroplasts were annotated using the STRING database. The interaction network of several defense regulators among these genes is presented in the right panel.

To identify the gene expression patterns and biological processes that respond uniquely or commonly to viral resistance in the mitochondria and chloroplasts of all three plant species, GO analysis of DEGs was performed using DAVID. First, the common genes were identified in the list of DEGs among the three species (Figure 2). The vast majority (approximately 90%) of these genes are uniquely associated with chloroplasts and mitochondria in the host defense against viral infection and replication. These processes, including plant-pathogen interactions, virus resistance, response to oxidative stress, response to hormonal signals, calcium-binding proteins, apoptosis, glycosylation, protein ubiquitination, chloroplast RNA binding, photosynthesis, and related processes, are directly involved in defense mechanisms against viral infections within mitochondria and chloroplasts. Additionally, processes such as biosynthesis of secondary metabolites, cellular response to hypoxia, response to wounding, circadian rhythm regulation, cell wall biogenesis and organization, plant growth regulation and development processes, lipid metabolic processes, and unsaturated fatty acid biosynthetic processes are indirectly related to understanding how mitochondrial and chloroplast functions contribute to plant defense against viral infections. Eventually, among these common DEGs, 47 important genes (including 38 downregulated and 9 upregulated genes) that interacted with each other were employed for GO analysis using the STRING database. Finally, the network predicting the potential role of these genes was drawn using the Genemania algorithm (Figure 4). Examination of this network also identified downregulated DEGs that were expressed directly in chloroplasts, including *POR, STR16, PLR1, PLGG1, PSBY, DXS, APO2, BETA, OHASE_2, CHLI2, PRXIIE, PTAC14, FAD2, PTAC14, PRXIIE, PTAC14,* and *FAD2,* as well as in mitochondria, such as *GLDP1*. A comprehensive analysis of these DEGs identified several defense regulators, including *PORA/B/C, ASP2T, NAC104, ZAT6, CHLI2,* and *CML*, as well as several genes with unknown functions, such as *AT2G41640*, and *AT3G57380* as promising candidates for further investigation.

### 3.5 WGCNA and module identification

We constructed a co-expression network analysis utilizing the WGCNA package on the DEGs to reveal interconnected expression patterns among the samples analyzed. To optimize the network structure and minimize weak correlations, we applied a soft-thresholding method with a scale-free model-fitting index of *R^2^* < 0.8, which ensured a high average interaction count among the genes. Power (*β*) values of 20, 20, and 10 were chosen for the *Arabidopsis*, tobacco, and rice datasets, respectively, to produce a dendrogram (Supplementary Figure 1). Thus, DEGs were grouped based on the dynamic tree pruning algorithm into seven modules for the *Arabidopsis* dataset with sizes ranging from 33 to 646 genes per module, six modules for the tobacco dataset with sizes ranging from 139 to 1,420 genes per module, and six modules for rice with sizes ranging from 32 to 189 genes per module were grouped (Table 2; Supplementary Figure 2). The gray module index genes that were not significantly co-expressed with any other group of genes were considered exceptions (Supplementary Figure 2).

**Table 2:** Modules of four types of co-expression analysis on each dataset.

Datasets	Module name	Total modules	Total hubs
*Arabidopsis*	Blue	536	210
	Brown	196	
	Green	55	
	Red	48	
	Turquois	646	
	Yellow	162	
	Black	33	
Tobacco	Blue	1,355	180
	Brown	266	
	Green	163	
	Red	139	
	Turquois	1,420	
	Yellow	259	
Rice	Blue	185	178
	Turquois	189	
	Brown	65	
	Green	37	
	Red	32	
	Yellow	48	

**Figure 5:**
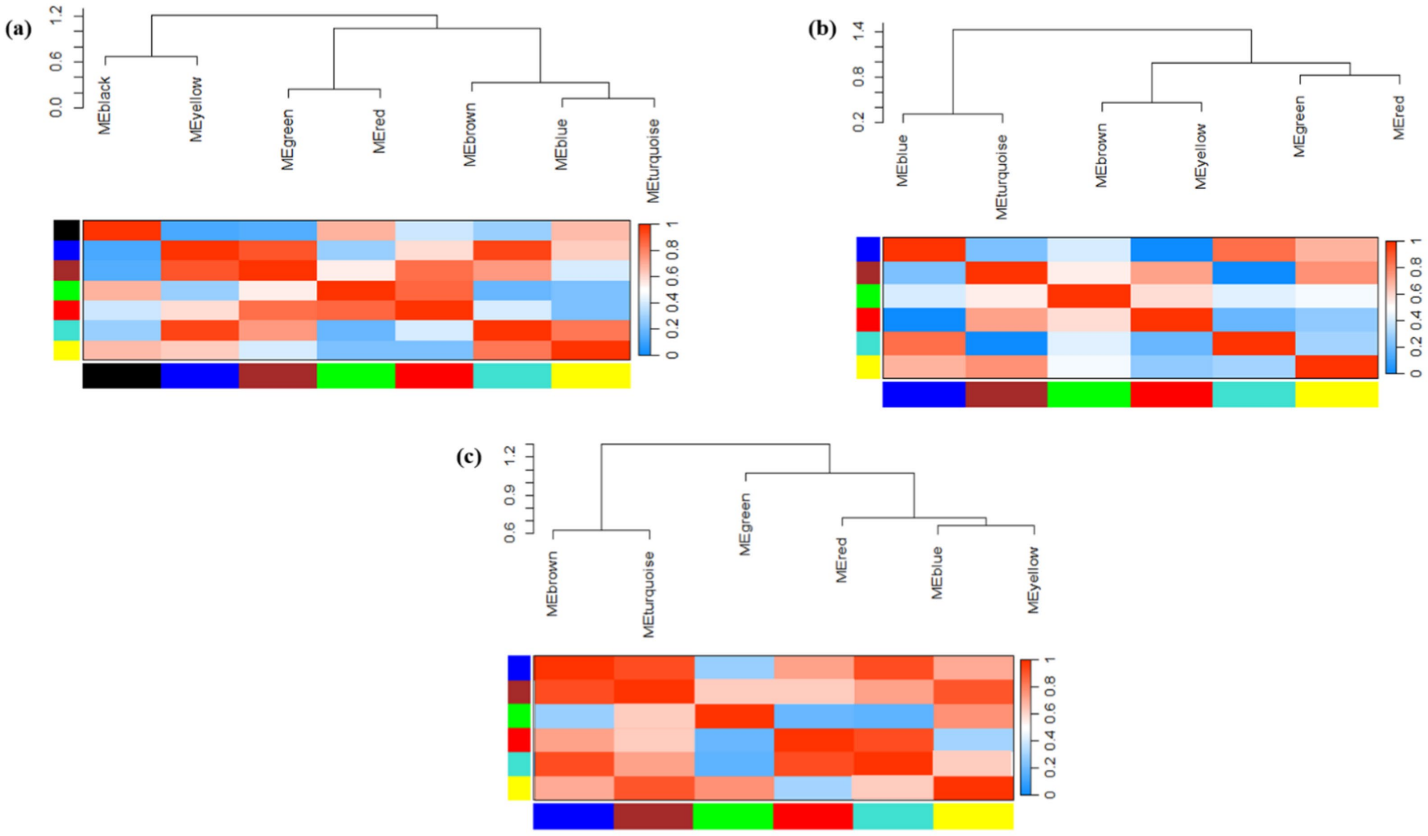
Hierarchical clustering dendrogram and heat map showing module eigengene adjacency in **(a)** *Arabidopsis* **(b)** tobacco and **(c)** rice plants challenged by viruses. In the heatmap, red color showed a positive correlation, whereas blue color showed a negative correlation.

**Figure 6:**
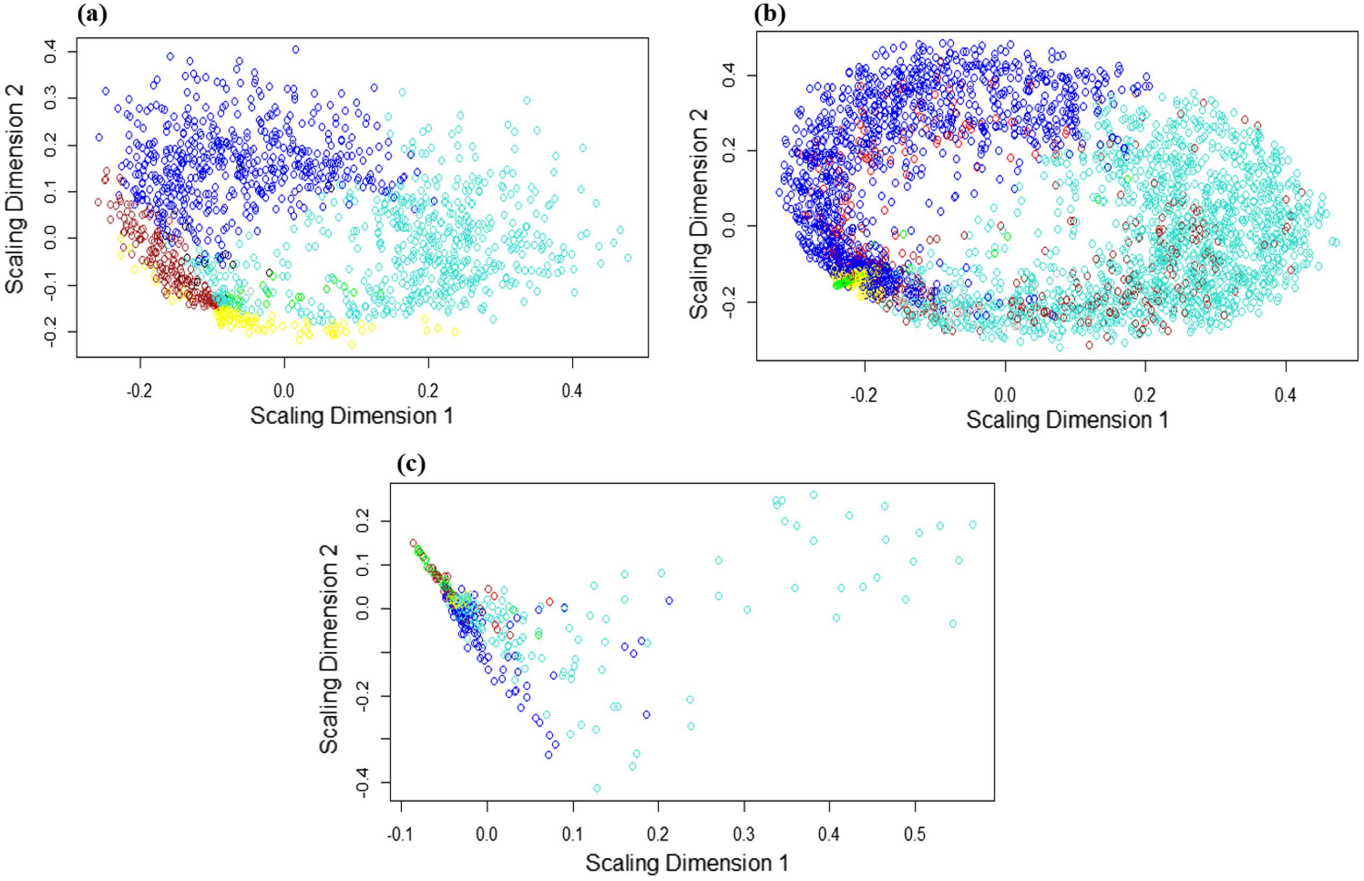
Multidimensional scaling (MDS) expansion plot of genes located in each module of **(a)** *Arabidopsis* **(b)** tobacco and **(c)** rice plants. MDS plot showing the similarity of gene expression levels between separate modules. Genes in the separate modules are labeled with distinct colors. The axes represent the scaling dimensions of variation among the genes.

Gene co-expression networks (GCN) are composed of genes that have similar profiles and are highly correlated with each other. As the hierarchical clustering shows, we obtained two clusters, including 5 subclusters for the *Arabidopsis* dataset, 4 subclusters for the tobacco dataset, and 4 subclusters for rice (Figure 5). According to the multidimensional scaling (MDS) of the *Arabidopsis* dataset, the genes in the modules, including green, yellow, turquoise, as well as brown and red genes, indicated similar expression patterns (Figure 6a). In the tobacco dataset, genes in the red, green, and brown modules indicated a similar expression patterns (Figure 6b). Finally, in the rice dataset, genes in most modules showed similar expression patterns (Figure 6c).

In the adjacency matrix heat map, the variance gradient from black to yellow indicates the connectivity of genes for separate modules from strong to weak, whereas red highlights indicate the strongest modules in the dataset. In the heatmap related to the *Arabidopsis* dataset (Figure 5a), the brown-blue and yellow-turquois modules, in the tobacco dataset (Figure 5b), the brown-green-red modules, and in the rice dataset (Figure 5c), the brown-blue and red-turquois indicated the strongest gene clustering based on TOM dissimilarity metrics. Additionally, based on the TOM, darker colors indicate lower overlap, whereas progressively lighter colors indicate a greater overlap. The light-colored blocks are along the diagonal of the modules (Supplementary Figure 3).

In conclusion, KEGG pathway annotation of the modules was performed separately for the three plant species separately (Supplementary Table S2). In each of the three plant species, most modules revealed significant metabolic pathways, such as biosynthesis of secondary metabolites, photosynthesis, carbon metabolism, glyoxylate/dicarboxylate, and glutathione metabolism. Glutathione is a key antioxidant that helps mitigate oxidative stress during viral infections, whereas secondary metabolites can directly inhibit viral replication or enhance host resistance. The involvement of phenylpropanoid biosynthesis in the blue module is particularly noteworthy, as it suggests a coordinated response involving metabolic reprogramming, protein synthesis, and signaling in response to viral infections. The presence of DEGs related to plant-pathogen interactions in turquoise (14 genes), blue (12 genes), and brown (7 genes) modules in *Arabidopsis*, as well as in the turquoise (6 genes) module of tobacco, indicates that these metabolic processes may play a direct role in the mitochondrial and chloroplast immune mechanisms against viral infections. However, no module related to plant-pathogen interactions has been identified in rice plants challenged by viral infection. These pathways are essential for plant metabolism and play a crucial role in energy production and stress responses during viral infections, suggesting that genes involved in their synthesis may enhance plant resilience to viral attacks.

### 3.6 Identification of hub genes and enrichment analysis

**Figure 7:**
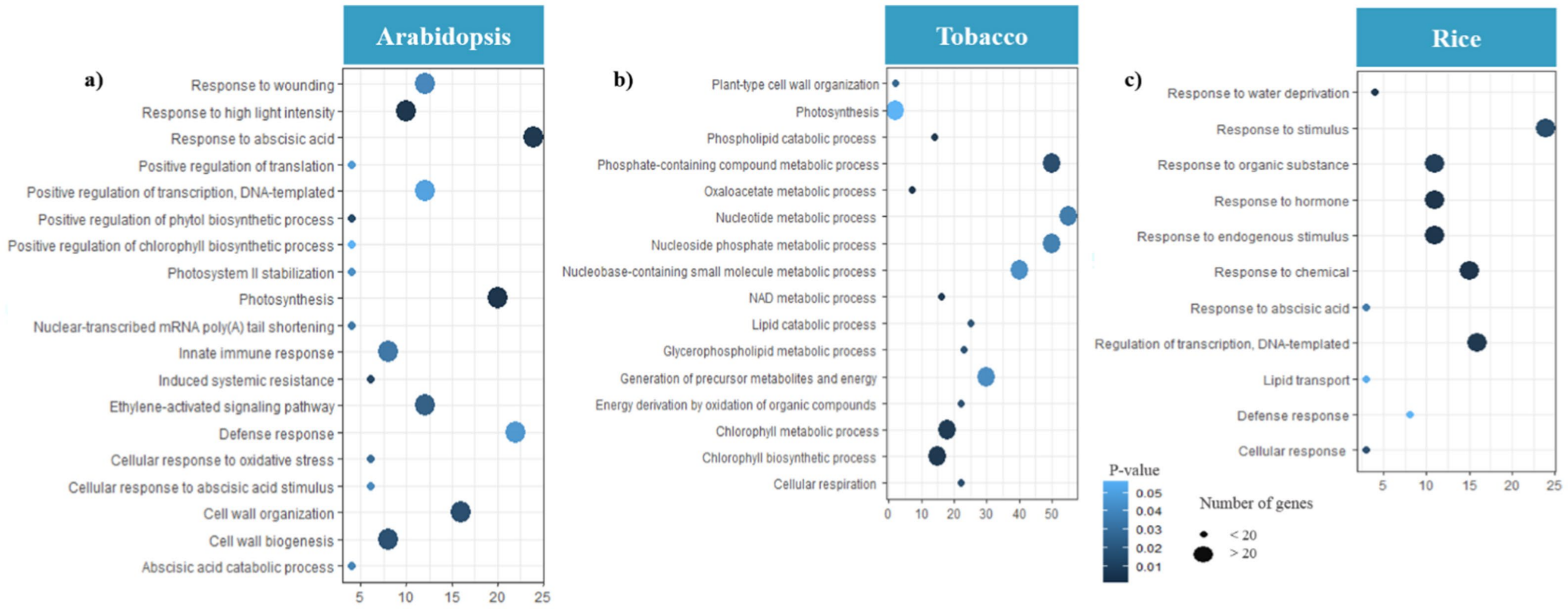
Gene Ontology (GO) analysis of hub genes. Distribution of GO enrichment of BP for **(a)** *Arabidopsis* **(b)** tobacco and **(c)** rice plants during viral infection. The color of each bubble was determined based on the *p-*value of the discovered pathways (*p-*value ≤ 0.05). The bubble size represents the number of hub genes enriched in these pathways. The X-axis indicates the number of genes and the Y-axis indicates enriched GO terms. The Figures were drawn using R language and ggplot packages.

To screen hub genes, we established a network of co-expressed modules using the Cytoscape software. We identified 210, 180, and 178 hub genes in *Arabidopsis*, tobacco, and rice plants, respectively, during viral infection, respectively (Supplementary Table S3). Through GO enrichment analysis, we elucidated the key mechanisms involved in chloroplast and mitochondrial responses in host defense and viral genome replication (Figure 7). The hub genes associated with *Arabidopsis* modules were highly expressed in defense response, innate immune response, induced systemic resistance (ISR), and cellular response to oxidative stress. In addition, functions such as response to ABA, photosynthesis, cell wall organization, ethylene-activated signaling pathways, and wounding indirectly influence the ability of plants to mount an effective defense against viral infections (Figure 7a). In tobacco, focusing on pathways such as energy derivation by oxidation of organic compounds, cellular respiration, nucleoside phosphate, and nucleotide metabolic processes provides a robust framework for understanding how mitochondrial and chloroplast-related genes contribute to both viral genome replication and host defenses against viral infections (Figure 7b). The role of biological processes, including chlorophyll metabolism, photosynthesis, phosphate-containing compound metabolism, glycerophospholipid metabolism, and oxaloacetate metabolism, in directly combating viral infections is minimal compared to the metabolic pathways directly involved in energy production and nucleotide synthesis (Figure 7b). In rice, the pathways of defense response, regulation of transcription, DNA-templated, and cellular response directly emphasize the essential roles of mitochondrial and chloroplast genes in virulence (Figure 7c).

### 3.7 Identification of TFs and TRs involved in defense responses

TFs are essential proteins that regulate DEGs expression by binding to specific DNA sequences, thereby controlling the transcriptional activities of various genes. They play pivotal roles in both viral pathogenesis and host defense mechanisms during viral infection, influencing the expression of viral and host genes through complex regulatory networks.

**Figure 8:**
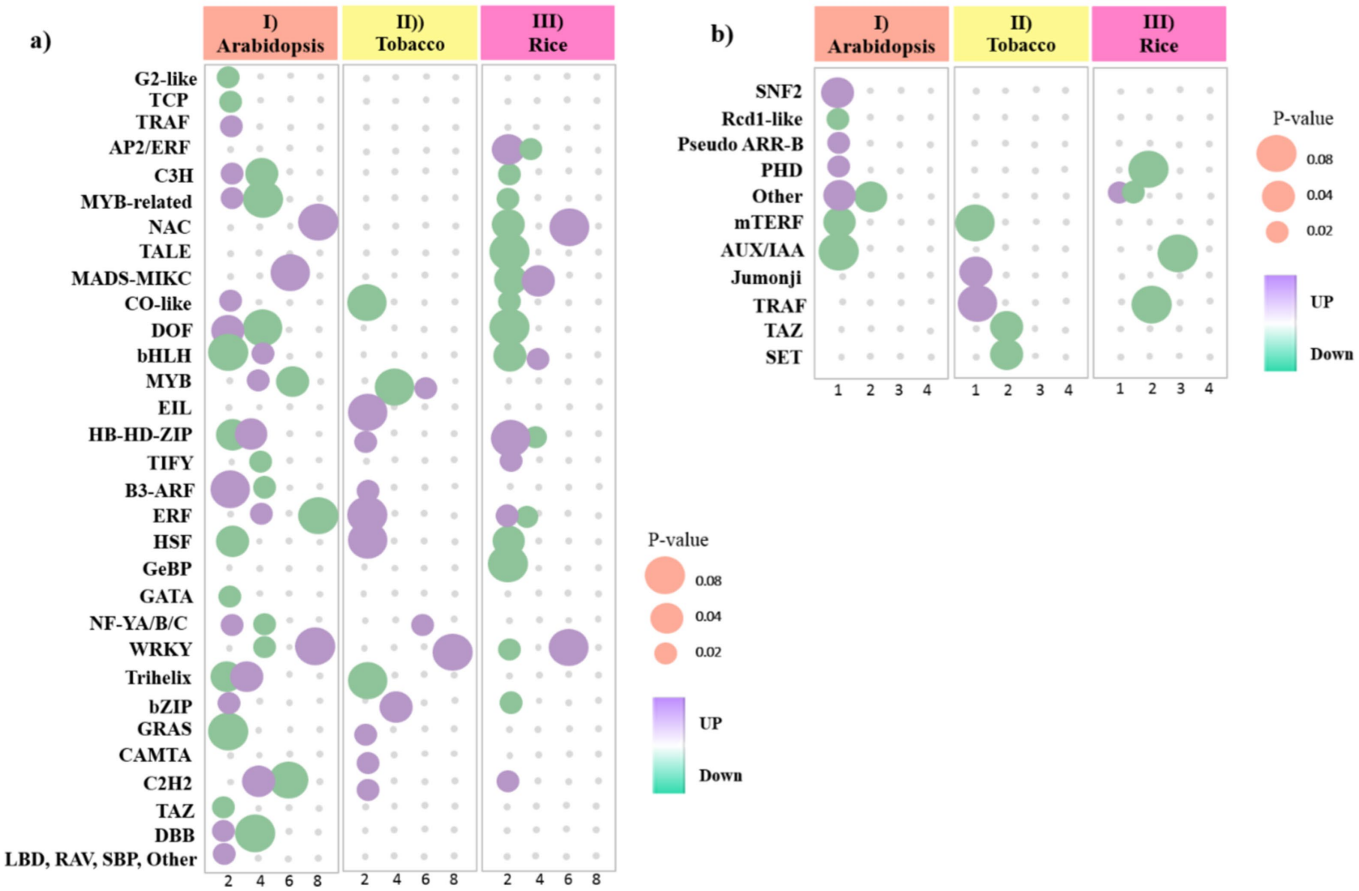
Bubble heat plot showing further analysis of DEGs between (I) *Arabidopsis* (II) tobacco and (III) rice plants during viral infection. **(a)** Distribution of upregulated and downregulated TF families detected in DEGs. **(b)** Distribution of upregulated and downregulated TR families detected in DEGs. Bubbles color represents the upregulated and downregulated genes. The X-axis indicates to the number of genes. The Y-axis represents the TF/TR families. The Size of the bubble was determined based on the *p-*value of the discovered genes of each family (*p*-value ≤ 0.05).

We identified 129 genes from 31 unique TF families in the *Arabidopsis* dataset, among which C2H2, Dof, ERF, HD-ZIP, MIKC-MADS, MYB, NAC, and WRKY families were the most abundant TF families, and most of them were downregulated (Figure 8a; Supplementary Table S4). According to the results, a total of 38 genes from 13 TF families were found in the tobacco dataset, most of which were upregulated, and only one Trihelix member, one EIL member, and two MYB members were downregulated. Also, among them, MYB, NF-YA, and WRKY families were the most abundant TF families (Figure 8a; Supplementary Table S4). Moreover, a total of 42 genes from 17 TF families in the rice dataset were downregulated, and among them, NAC, bHLH, and WRKY families were the most abundant TF families (Figure 8a; Supplementary Table S4).

TRs function as transcriptional activators and repressors, enabling plants to activate defense mechanisms and coordinate regulatory networks to respond adaptively to viral infection. We identified 12 genes from eight TR families in the *Arabidopsis* dataset. Among the TRs related to the *Arabidopsis* data, most of them were downregulated, except for the three families PHD, SNF2, and Pseudo ARR-B (Figure 8b; Supplementary Table S5). Additionally, we identified 7 genes out of the 5 TR families in the tobacco dataset. Among the relevant TRs, most of them were downregulated, except for the TRAF and Jumonji families (Figure 8b; Supplementary Table S5). In rice, 10 genes from four families, including four AUX/IAA members, were identified. However, the number of TRs with upregulated was less than TRs with downregulated, and only one DEG from other families had upregulated (Figure 8b; Supplementary Table S5). These findings highlight the dynamic interplay between TFs and viral infections, underscoring their critical role in modulating host defense strategies.

### 3.8 Prediction of miRNAs targets

**Figure 9:**
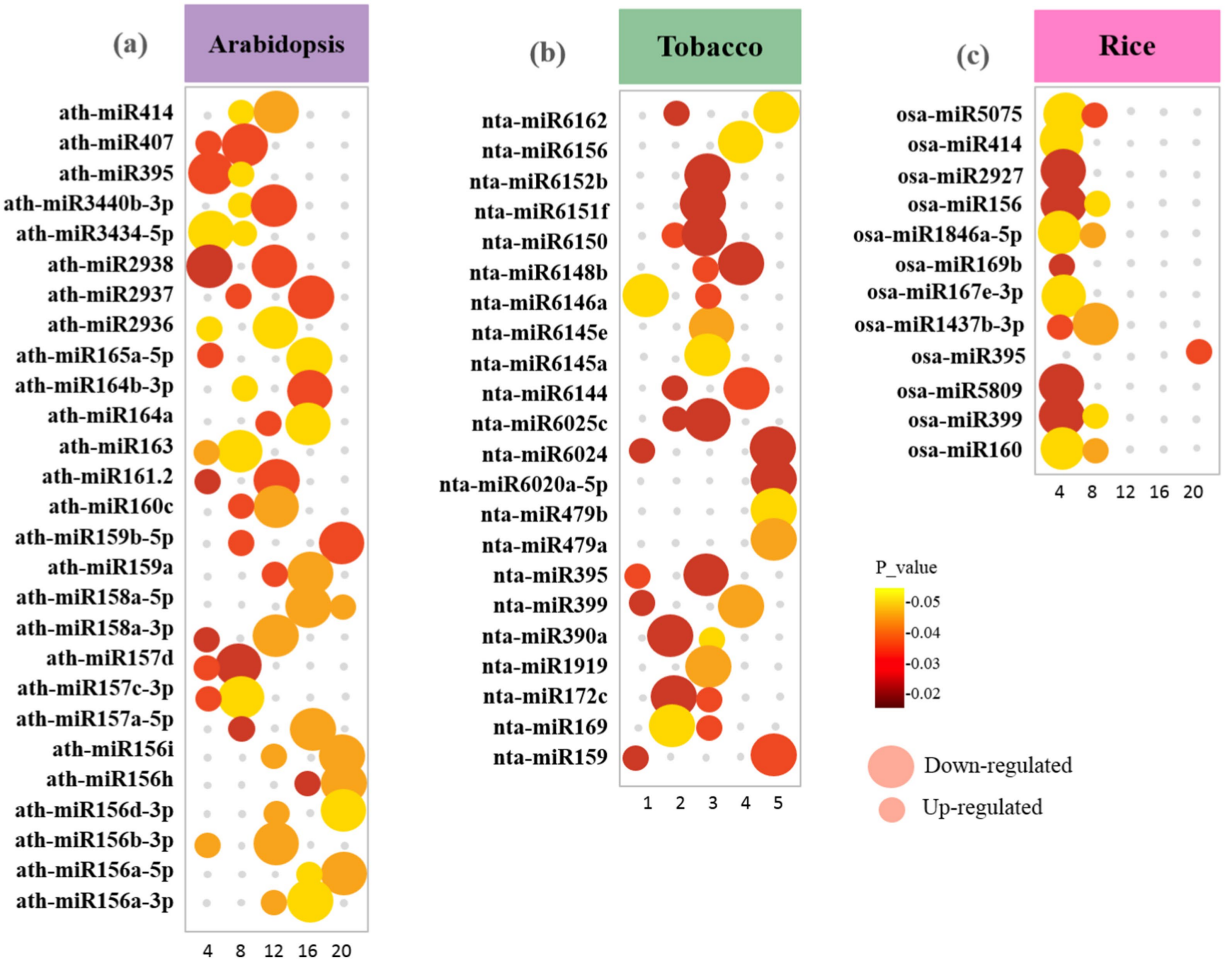
Bubble plot showing the distribution of the highest-scoring miRNA families identified in upregulated and downregulated DEGs between **(a)** *Arabidopsis* **(b)** tobacco and **(c)** rice plants during viral infection. The size of the bubbles represents upregulated and downregulated genes. The X-axis refers to the number of genes. The Y-axis shows miRNA families. Bubbles color was determined based on the *p-*value of the discovered genes of each family (*p-*value ≤ 0.05).

Statistical analyses have led to the identification of 1820 conserved miRNAs belonging to 427 families in *Arabidopsis* DEGs, 216 miRNAs belonging to 159 families in tobacco DEGs, and 64 miRNAs belonging to 300 families in rice DEGs (Supplementary Table S6). A comparative analysis showed that the *Arabidopsis* dataset had the highest number of DEmiRNAs (769 upregulated and 1,051downregulated), followed by the tobacco dataset (62 upregulated and 154 downregulated), and then the rice dataset (26 upregulated and 38 downregulated). Investigation of the datasets resulted in a total number of 12 DEmiRNAs that were common among all datasets (miR395a, miR1569, miR160, miR171, miR169c, miR395b, miR395c, miR827, miR166f, miR399e, miR172a, miR319b, miR399f, and miR167b). *Arabidopsis* and rice DEGs shared a maximum of 25 DEmiRNAs with conserved expression patterns, followed by the *Arabidopsis* and tobacco datasets of 24 DEmiRNAs, and finally the tobacco and rice datasets of 5 DEmiRNAs (Supplementary Table S6). Several miRNA families contain only one member. The abundance of different members in the same miRNAs family varied significantly. The expression levels of several miRNAs, miR390, miR1428, miR1846a-5p, miR2924, miR414, miR1437b-3p, miR172c, miR156a-5p, and miR156h, were relatively high (Figure 9).

**Figure 10:**
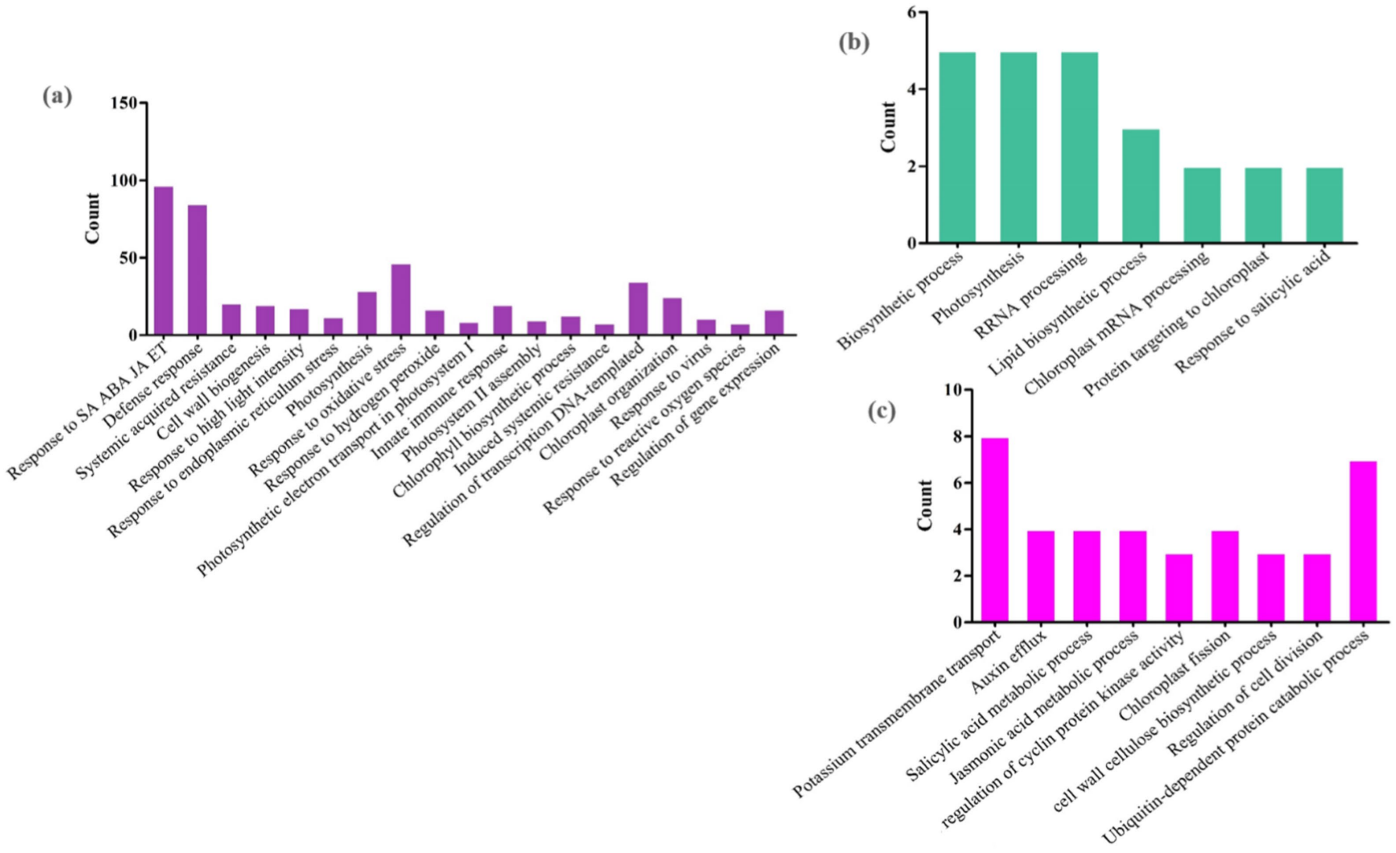
Possible functions of miRNA target genes in **(a)** *Arabidopsis* **(b)** tobacco and **(c)** rice plants during viral infections. The horizontal axis represents GO classification, and the vertical axis represents the number of DEGs. The miRNAs with a significant number of genes are presented here.

The possible functions of mitochondria/chloroplast-associated DEmiRNA-targeted genes in viral genome replication and host immune response against viral infections were identified by GO analysis (Figure 10). We found that all target DEGs of *Arabidopsis* play a role in response to viruses, defense responses, systemic acquired resistance (SAR), hormone-mediated defense, innate immune response, response to oxidative stress, plant-type hypersensitive response, induced systemic resistance (ISR), and chloroplast organization (Figure 10a). In tobacco plants, genes responsive to photosynthesis, chloroplast mRNA processing, response to salicylic acid, tricarboxylic acid cycle, rRNA processing, and protein targeting to chloroplasts emphasize the interconnectedness of chloroplast functions and plant defense against viral threats (Figure 10b). In addition, genes involved in mRNA processing, SA/JA metabolic process, chloroplast fission, negative regulation of serine/threonine kinase activity, and ABA-activated signaling pathway were identified to be targeted by miRNAs in rice plants (Figure 10c). These terms highlight key metabolic processes and regulatory mechanisms that are likely to be essential for understanding how mitochondrial and chloroplast functions contribute to both viral replication and host defense strategies.

### 3.9 Cis-acting elements analysis of DEGs

The 1,000 bp upstream flanking region of the DEGs was used to predict conserved cis-regulatory elements (CREs). For the *Arabidopsis*, tobacco, and rice datasets, the MEME analysis identified 11 crucial cis-acting elements (Supplementary Table S7). Separate analyses of the three plant species suggested that Other C4 zinc finger-type factors, GATA, MADS-box, BBR/BPC, C2H2 zinc finger factors, AP2/EREBP, DOF, FOX, and ERF, play key roles in transcriptional activation and could form promoters that drive transgene expression (Supplementary Table S7).

GOMO analysis of the motifs identified using MEME revealed several interesting BFs (Supplementary Tables S8, S9). GO term analysis of *Arabidopsis* DEGs indicated that these motifs were involved in TF activity, the transmembrane receptor protein tyrosine kinase signaling pathway, regulation of DNA-dependent transcription, mitochondrial transport, protein amino acid phosphorylation, and translation. Moreover, these motifs were involved in the MFs of a structural constituent of ribosomes, protein binding, TF, and protein serine/threonine kinase activities (Supplementary Table S8). GO term analysis of tobacco DEGs showed that the motifs were mainly involved in the transmembrane receptor protein tyrosine kinase signaling pathway and protein amino acid phosphorylation (Supplementary Table S8). In addition, these motifs were involved in MFs, including transcription factor activity, protein binding, and protein serine/threonine kinase activity (Supplementary Table S8). Finally, GO enrichment analysis of rice motifs responsive to viral stress was performed. These processes include the regulation of transcription of DNA-dependent, transmembrane receptor protein tyrosine kinase signaling pathway, protein amino acid phosphorylation, and flower development. The analysis of MF revealed that these motifs were involved in TF activity, protein binding, and ATP binding (Supplementary Table S8).

Because the motif is a sequence pattern that occurs repeatedly in a group of DNA sequences, determining the most abundant sequences related to mitochondria/chloroplasts was considered in this study. In *Arabidopsis*, four chloroplast-related motifs (Motifs 4, 5, 9, and 10) and two mitochondrion-related motifs (Motifs 5 and 9) are induced during viral infection. Moreover, five chloroplast-related motifs (Motifs 3, 4, 5, 6, and 8) were induced in the virus-infected rice plants. No chloroplast/mitochondrion-related motifs were found to be induced in tobacco plants challenged by viral infection (Supplementary Table S8).

### 3.10 PPI and selection of key hub genes

**Figure 11:**
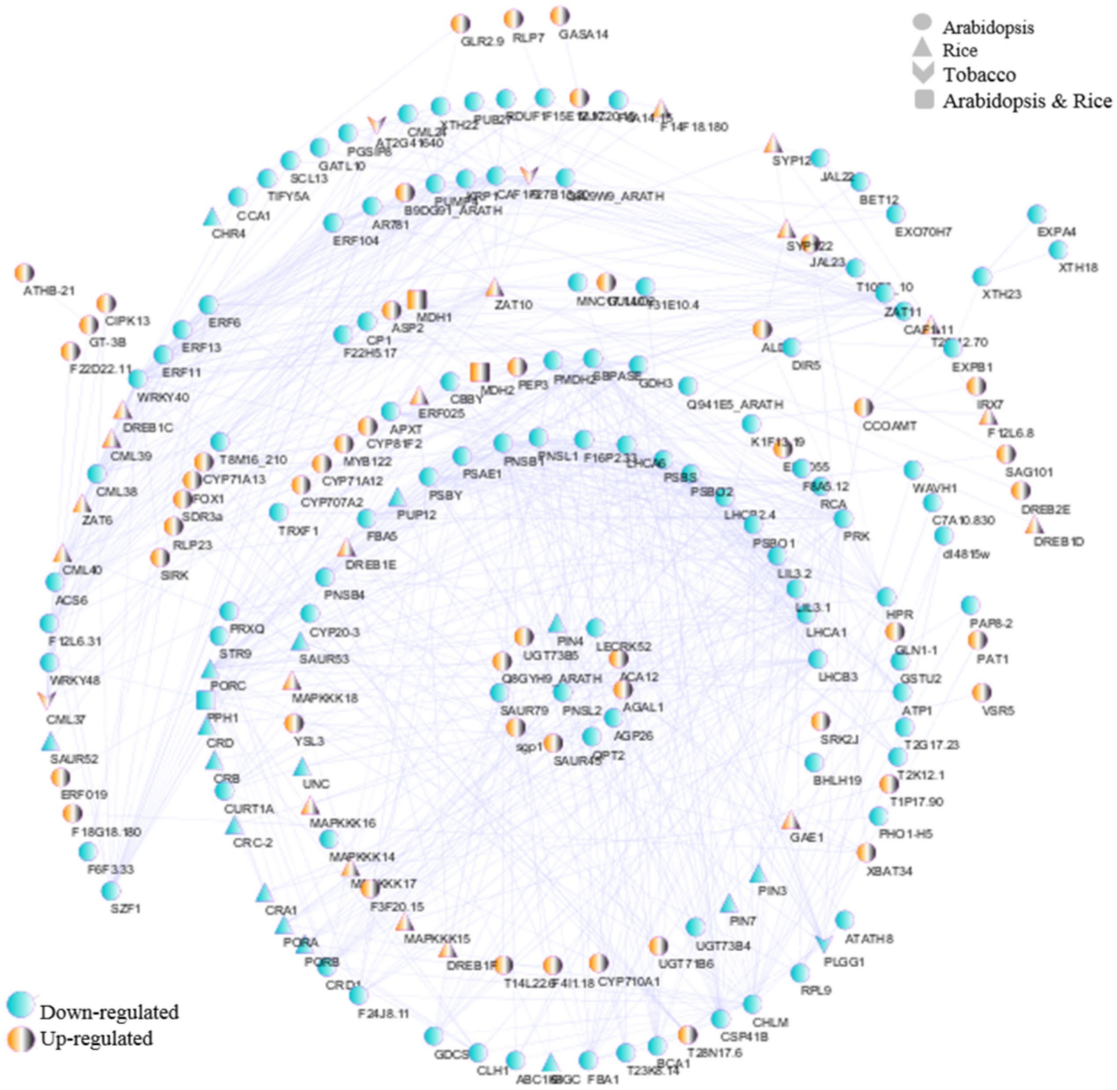
PPI network analysis was performed using the STRING database and Cytoscape software. Network of interactions among hub genes in *Arabidopsis*, tobacco, and rice datasets. The yellow and blue nodes represent upregulated and downregulated genes, respectively.

To reveal the interactions of mitochondrial/chloroplast-related genes against viral infections in *Arabidopsis*, tobacco, and rice plants, PPI networks were drawn according to the identified DEGs. Initially, to comprehend the degree of conservation in the proteins interactions in each of the three species against viral infection, a network of all identified hub genes from each of the three datasets was drawn with 88 nodes connected with 661 edges (Figure 11). In this network for the tobacco dataset *MDH1*, and *MDH2* hub genes with upregulated, and *PPH1* gene with downregulation were identified. In the rice dataset, among the hub genes with the most interactions, *CML40, DREB1C, DREB1E,* and *MAPKKK17* were upregulated, and *CRB, CRA1, PORB, PORA,* and *PORC* were downregulated. In the *Arabidopsis* dataset, hub genes with the most interactions, including *ALD1, CYP81F2, ASP2, CYP71A12, CYP71A13,* and *FOX1* were upregulated, and *CHLM, LHCA1, LHCA6, CRD1, LHCB3, CSP41B, APXT, BCA1, LIL3.1, PNSB1,* and *ERF6* were downregulated.

In general, it is possible to introduce downregulated hub genes related to chloroplasts for further investigation of defense response pathways and virus replication, including *POR, RCA, PSBY, PSBS, LIL3.1, dl4815w, PAP8-2, RPL9, PSBO1, STR16, ABC1K8, MNC17.140, APO2, LHCA/B, ATP1, TAT1, CYP20-3, CRD1, SBPASE, PSBO2, PSAE1, CHLM, CURT1A, PLGG1, PRK, PRXQ, TRXF1, BCA1, APXT, PNSB4, CHLI, PNSB1,* and *STR9* (Figure 11). In addition, upregulated hub genes such as *PAT1* were associated with chloroplasts (Figure 11). Finally, the hub genes *CML*, *PUMP4, GDH3, GDCST, NDA2, T31E10.4,* and *CBSX3* were associated with mitochondria (Figure 11). These findings underscore the need to further investigate the intricate molecular mechanisms by which mitochondrial/chloroplast-related genes contribute to plant resilience against viral infections, potentially revealing novel targets for enhancing viral resistance in crops.

### 3.11 Leave-one-out cross validation of meta-analysis

**Figure 12:**
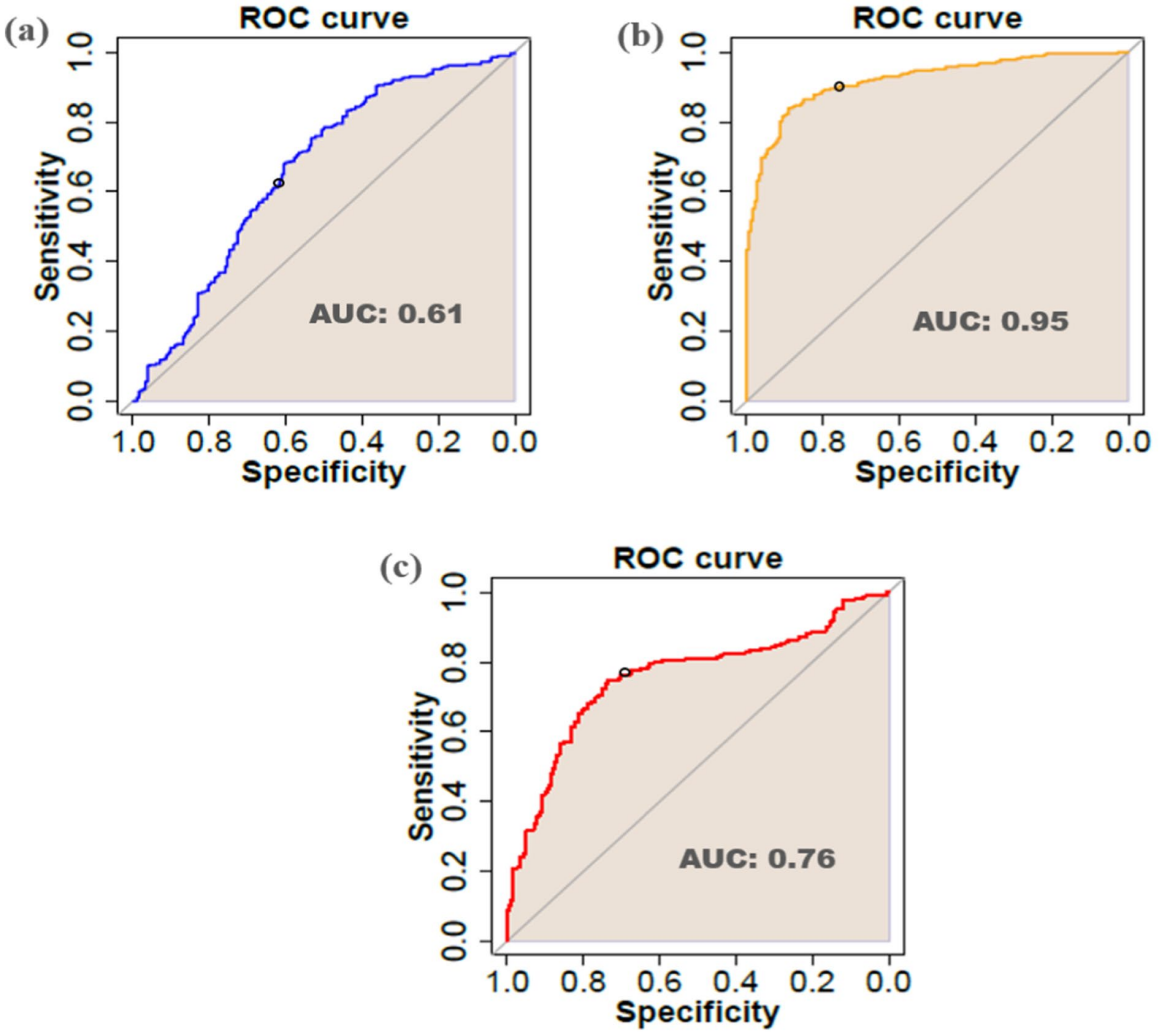
Receiver-operating characteristic (ROC) curve and AUC of DEGs identified in **(a)** *Arabidopsis* **(b)** tobacco, and **(c)** rice datasets.

To validate the DEGs efficiency in viral infections, the LOOCV approach was applied. According to the results (Figure 12), the DEGs identified for the *Arabidopsis*, tobacco, and rice datasets had AUC values of 0.61, 0.95, and 0.76, respectively.

## 4 Discussion

### 4.1 The role of host molecular function in regulating the viral replication process in chloroplast/mitochondria

Analysis of viral replication processes in chloroplasts has revealed intricate interactions between host proteins and viral components, which are essential for successful infection and replication. Various host factors are significant for the assembly and function of viral replication complexes (VRCs) within chloroplasts, particularly in plant systems, such as rice, tobacco, and *Arabidopsis* (Budziszewska and Obrepalska-Steplowska, 2018; Bwalya and Kim, 2023). The identification of these factors can be accomplished through genome-wide screening, which reveals insights into how viruses manipulate the host cellular machinery. Chloroplast membranes are critical sites of viral replication (Wu et al., 2024). For instance, TMV and TuMV utilize the chloroplast protein phosphoglycerate kinase (ChlPGK) to facilitate the translocation of viral ribonucleoprotein complexes across membranes into the stroma, promoting VRC assembly near thylakoids (Bhattacharyya and Chakraborty, 2018). Replication complexes are often associated with peripheral vesicles and cytoplasmic invaginations on the outer chloroplast membrane, where they engage in complex interactions with host proteins involved in lipid metabolism and transport (Bwalya and Kim, 2023; Cheng et al., 2013). Research has indicated that viruses exploit various host cellular structures, including peripheral vesicles and cytoplasmic invaginations (CIs), to enhance their replication efficiency. Moreover, viruses employ a variety of host enzymes such as RNA helicases, translation factors, methyltransferases, and chaperones to aid in their replication. These host proteins not only assist in the proper folding of viral RNA but also play roles in the recruitment of viral components to replication sites. The formation of VRCs is closely linked to lipid metabolism; for example, chloroplast membrane lipids are associated with viral proteins, aiding the localization of viral RNA for replication (Geng et al., 2017; Hyodo and Okuno, 2014). Moreover, oxidative stress management is crucial during viral infections. Antioxidant enzymes, such as glutathione transferases, help mitigate the reactive oxygen species (ROS) generated during replication, thus promoting efficient viral RNA synthesis (Huang et al., 2017). The interaction of viral components with host ATP synthase and other chloroplast proteins underscores the complexity of these relationships in *N. benthamiana* and *A. thaliana* plants infected with TMV (Megias et al., 2018). Overall, understanding these host-virus interactions provides insight into where viruses leverage host factors for efficient replication while also triggering stress responses that must be manipulated to enhance plant resistance.

### 4.2 Biological processes of chloroplast/mitochondria affect plant defense against viruses

Chloroplast is a vital organelle in plants and is primarily known for its role in photosynthesis, where it provides energy and carbon. However, during viral infections, plant defense mechanisms can compromise photosynthesis and other anabolic processes, thereby limiting the availability of organic carbon to pathogens (Bwalya and Kim, 2023; Serrano et al., 2016). This reduction in photosynthetic efficiency is often manifested through symptoms, such as leaf chlorosis and necrosis, which are typical of plant RNA viral infections. Additionally, chloroplasts serve as a significant origin of various defense-related signals and are intricately linked to the initiation of effector-triggered immunity (Bhattacharyya and Chakraborty, 2018; He et al., 2021). Specifically, it induces plant responses to viruses through the production of secondary metabolites, including calcium, ROS, chlorophyll proteins, and defense-related hormones, which are critical for host immunity. This dual role of the chloroplast underscores its importance not only in energy production but also as a key player in the immune response of plants (Farooq et al., 2024; Gu et al., 2024). Thus, understanding the biological pathways associated with chloroplast function could provide insights into strategies to enhancing viral resistance in plants.

SA is a small phenolic composition that significantly contributes to the defense mechanisms of plants against biotrophic parasites, and is crucial for the establishment of SAR. Pathogen-induced SA is mainly produced via the isochorismate process in chloroplasts (Seyfferth and Tsuda, 2014). Interestingly, isochorismate synthase was the most downregulated DEG in the tobacco plants. Disruption of the SA biosynthetic pathway severely impairs plant resistance to viral infection. In contrast, application of SA mainly enhances plant basal immunity, thereby delaying the onset of viral infections and disease progression (Alazem and Lin, 2015). For example, overexpression of *PAP2*, a chloroplast-localized protein, resulted in upregulation of the SA pathway and resistance to SMV infection (Widyasari et al., 2022). Another chloroplast-localized protein, called the calcium-sensing receptor, has been discovered as a critical regulator that operates upstream of SA accumulation, thereby linking chloroplast activity to immune responses in both the cytoplasm and the nucleus of *A. thaliana* (Nomura et al., 2012).

JA plays a significant role in enhancing immune defenses during compatible plant-virus interactions. JA is an oxylipin that originates from polyunsaturated fatty acids, specifically *α*-linolenic acid (18:3) (α-LeA), released from the chloroplast membranes. Thus, α-LeA is catalyzed by lipoxygenase (*LOX*) to yield 12-oxo-phytodienoic acid (*OPDA*) (Soltani et al., 2023). This pathway underscores the integral connection between chloroplast function and hormonal signaling in plants. Research indicates that silencing of Coronatine Insensitive 1 (COI1), a critical gene in the JA signaling pathway, leads to an accelerated manifestation of symptoms associated with co-infection by CaLCuV and PVA/X. This silencing also results in increased viral titers during the early stages of infection, highlighting the importance of JA in regulating plant responses to viral pathogens (García-Marcos et al., 2013). Upon detection of infection, JA is conjugated to isoleucine by the enzyme JAR1 to form jasmonoyl-isoleucine (JA-Ile), which acts as a bioactive form of JA. When JA-Ile binds to COI1, it triggers the degradation of JAZ proteins, which are repressors of JA signaling. This process allows for the expression of defense genes that are essential for combating viral infections.

The ABA biosynthesis pathway is also involved in antiviral immune responses in plants (He et al., 2021). Recent studies have shown that ABA enhances the expression of antiviral RNA-silencing genes in soybean and *A. thaliana*, which confers partial resistance against infection. For example, while ABA treatment improves resistance to TMV, research on rice has demonstrated that applying ABA can lead to increased susceptibility to viral infections by inhibiting JA biosynthesis and raising ROS production (Xie et al., 2018).

ROS are primarily generated in chloroplast thylakoids, specifically at the reaction centers of photosystems I (PSI) and II (PSII), where they play crucial roles in plant immune mechanisms against viral infections. The production of superoxide anions (O2−) through the photoreduction of molecular oxygen is a key process that leads to the formation of hydrogen peroxide (H2O2) through disproportionation reactions within thylakoid membranes. ROS production is not merely a byproduct of photosynthesis but serves as an essential signal for activating antiviral defense pathways in plants (Bwalya and Kim, 2023; Wu et al., 2017). When plants detect early viral infection, bursts of ROS and calcium ions trigger signaling cascades that regulate immune-related genes, establishing a retrograde signaling pathway from the chloroplasts to the nucleus (Bhattacharyya and Chakraborty, 2018; Medina-Puche et al., 2020). Furthermore, during both compatible and incompatible interactions with viruses, the accumulation of intracellular ROS can be observed, indicating their pivotal role in hypersensitivity responses (Hakmaoui et al., 2012). Chloroplast-derived ROS are particularly important for inducing hypersensitivity responses against viral pathogens, emphasizing the central role of chloroplasts in plant immunity (Zurbriggen et al., 2010). Additionally, specific chloroplast-localized proteins, such as N receptor-interacting protein 1 (NRIP1), have been implicated in mediating resistance against viruses, such as TMV. This resistance is often associated with ROS generation, further highlighting the multifaceted roles of chloroplasts in plant immunity (Caplan et al., 2008). Thus, it is evident that chloroplasts are not only sites of photosynthesis but also crucial hubs for ROS production and signaling in response to viral infections.

The MAPK signaling pathway plays a critical role in plant growth, response to environmental stresses, and pathogen attack (Meng and Zhang, 2013). Studies have indicated that two acidic endochitinase genes linked to pathogenesis-related protein PRs are induced in tobacco plants following infection by TMV, highlighting the significance of glyoxylate and dicarboxylate metabolism in plant defense mechanisms (Zhao et al., 2019). Viral infections typically result in symptoms, such as leaf chlorosis and necrosis, which can negatively affect chloroplast photosynthetic activity, further emphasizing the importance of MAPK pathways in mediating plant responses to viral threats.

GO enrichment analysis identified defense-related molecular pathways in different plant-virus interactions. ATPase activity, a mitochondrion-related function, has been found in virus-infected *Arabidopsis* and plays a role in antiviral defense (Chivasa et al., 2009; Schaller and Oecking, 1999). Chloroplast-related processes are also altered, with the chloroplast being an important organelle in plant-virus interactions (Bhattacharyya and Chakraborty, 2018; Zhao et al., 2016). Studies have shown that *Arabidopsis* plants use SAR against viruses, such as CMV and PPV (Love et al., 2007; Mayers et al., 2005). Hypersensitive responses and JA-mediated resistance were also observed in plants challenged with TMV (Kim et al., 2010) and CaLCuV (Shen et al., 2014), respectively. Biological processes, such as responses to JA and ISR, are regulated in virus-infected *Arabidopsis* plants. These findings highlight the diverse defense mechanisms employed by plants to combat viral infections.

### 4.3 Key TFs in chloroplast and mitochondrial responses to viral infections

Interactions among host plant viruses have evolved complex mechanisms to cope with plant resistance strategies. TFs play significant roles in plant growth, innate immunity, hormonal synthesis, and antiviral processes. During plant-viral interactions, certain TFs enhance plant immunity to viral infections by activating the expression of downstream resistance genes. To enhance infection in plants, various viral proteins commonly target and inhibit the activity of plant transcription factors (Falak et al., 2021; Tian et al., 2024). Many TF families, including WRKY, bZIP, ERF, NAC, DOF, bHLH, and MYB, have been reported to play key roles in transcriptional reprogramming and plant resistance.

DOF proteins are plant TFs characterized by a highly conserved single Cys zinc finger domain, which is crucial for their DNA-binding function. DOF binds to the promoter of a gene and acts as a transcriptional activator or repressor to regulate the signaling networks involved in oxidative stress, JA/SA hormones, and plant defense. They can indirectly affect the ability of plants to respond to viral infections by regulating genes involved in photosynthesis and mitochondrial/chloroplast metabolism. For example, the zinc finger domain of DOF can interact with the HSP70 promoter and inhibit the transcription of the *HSP70* gene and TMV viral infection factors (Falak et al., 2021; Tian et al., 2024). However, there are limited studies on the function of DOF TFs in plant-viral interactions.

WRKY transcription factors are crucial regulators of plant immune mechanisms, particularly in the response to viral infection. WRKY proteins are involved in pathogen-associated molecular pattern (PAMP)-triggered immunity (PTI) and effector-triggered immunity (ETI). They modulate mitogen-activated protein kinases (MAPKs) to regulate plant defense responses. Additionally, WRKY transcription factors are known to interact with plant resistance (R) proteins during ETI responses (Falak et al., 2021; Sun et al., 2023). Recent studies have highlighted the role of *WRKY30*, a member of the WRKY family, in enhancing CMV resistance in *Arabidopsis thaliana*. Mutant plants lacking *WRKY30* exhibit increased susceptibility to CMV, characterized by elevated oxidative damage and compromised photosystem II (PSII). In contrast, transgenic plants overexpressing *WRKY30* showed enhanced resistance to CMV, indicating that *WRKY30* functions as a positive regulator. Brassinosteroids (BRs) have been shown to induce CMV resistance through signaling pathways that partially depend on *WRKY30*. This interaction underscores the multifaceted role of *WRKY30* not only in direct viral defense, but also in mediating responses to hormonal signals that enhance plant immunity. ROS accumulation is a key cellular response to viral stress, where *WRKY30* appears to modulate ROS levels and activate antioxidant systems to mitigate oxidative damage. Moreover, the involvement of WRKY in N gene-mediated resistance to TMV in *N. benthamiana* and *Arabidopsis* plants has been reported (Zou et al., 2019). Overall, these findings indicate that *WRKY30* is integral to the transcriptional reprogramming necessary for effective viral resistance, thereby contributing significantly to our understanding of plant-virus interactions and the regulatory networks involved in antiviral defense mechanisms.

Evidence suggests that NAC TFs can enhance or inhibit viral replication by interacting with viral proteins. Recent studies have demonstrated that specific NAC TFs, such as *NAC6, NAC35, NAC22, NAC54,* and *NAC70*, are significantly upregulated in response to viral infections such as CMV. This upregulation correlates with enhanced hypersensitive responses (HR), a form of programmed cell death that restricts the spread of pathogens (Siriwan et al., 2022). Mutation of NAC TF results in susceptibility to RSV, suggesting that NAC is necessary for viral resistance. This expression pattern indicates that these TFs are crucial for mediating plant responses to viral stress (Bian et al., 2020). Moreover, the replication initiator proteins of geminiviruses have been found to interact with NAC TFs, potentially disrupting host DNA replication and facilitating viral propagation. This highlights a dual role of NAC proteins: they not only regulate host defense mechanisms but may also be manipulated by viral proteins to enhance viral replication (Dasgupta et al., 2021).

The MYB family plays a crucial role in plant immune mechanisms against viral diseases, particularly through the regulation of pathogenesis-related (PR) proteins and other defense-related genes. These factors are modulated by various phytohormones such as JA/SA, which activate *PR* gene expression at infection sites, thereby triggering SAR and enhancing the plant’s ability to combat viral pathogens. For instance, overexpression of *OsMYB4* in tomato has been shown to confer protection against viral infections, highlighting the significance of MYB in activating defense responses (Dasgupta et al., 2021). Similarly, the MYB TF family has been demonstrated to be involved in antiviral responses, such as HR, in TMV-infected tobacco plants (Yang and Klessig, 1996). In *Arabidopsis*, the upregulation of a member of the MYB TF family, specifically *AtMYB96*, has been observed in response to CMV infection (Geri et al., 1999). In addition to their role in *PR* gene activation, MYB transcription factors are involved in complex signaling pathways that include interactions with other transcription factors, such as WRKYs. For example, the MYB domain-containing transcription factor activates *RPL10* transcription, which inhibits geminivirus replication (Siriwan et al., 2022). These mechanisms suggest that the MYB/RPL10 protein network may contribute to the recovery of virus-resistant cultivars after exposure to viral infections. Furthermore, studies have indicated that the expression of MYB proteins increases in response to specific viral strains, although variations may occur depending on the host cultivar and type of virus involved. These studies underscore the importance of understanding the TF-driven responses in developing resistant plant varieties through breeding programs. Overall, transcription factors are essential components of the plant immune response, orchestrating a multifaceted defense strategy against viral threats.

### 4.4 miRNAs targets in chloroplast and mitochondrial responses to viral infections

MicroRNAs are critical regulators of DEGs expression at the post-transcriptional level and affect a variety of processes, such as plant growth, molecular pathways, and responses to stress conditions. Plant viruses can modify the host miRNA pathway to target mitochondrial/chloroplast-related transcripts (Millar, 2020). The role of miRNAs in viral infection, especially in terms of their origin in organelles, such as chloroplasts and mitochondria, is an emerging area of research. While most miRNAs are produced in the nuclear genome, recent studies have revealed that some miRNAs can also originate from chloroplasts and mitochondria (Anand et al., 2024). For instance, evidence suggests that chloroplasts can influence miRNA biogenesis through retrograde signaling to the nucleus under stress conditions, thereby enhancing the production of nuclear-derived miRNAs (Crawford et al., 2018). This signaling is critical during pathogen attack, as chloroplasts are often the primary targets, leading to significant functional disruptions (Bhattacharyya et al., 2015). Despite these findings, the mechanisms underlying miRNA biogenesis in chloroplasts remain poorly understood compared with those in mitochondria, where extensive studies have documented the presence and roles of mitochondrial miRNAs (Anand et al., 2024).

RSV causes devastating viral diseases in plants, hijacks mitochondrial/chloroplast-related proteins during infection, and disrupts photosynthesis (Shi et al., 2016). The disruption of photosynthesis caused by RSV is likely caused by the upregulation of a specific miRNA that targets essential genes involved in the chloroplast zeaxanthin cycle, ultimately leading to impairment of both chloroplast structure and function (Yang et al., 2016). miRNAs not only serve as regulators of gene expression but also play a vital role in the complex interactions between plants and viruses. Their capacity to modulate hormonal signaling pathways and gene expression in response to pathogen attack underscores their potential as targets for enhancing viral resistance in crops (Zhou et al., 2016). Specific miRNAs, such as miRNA156, miRNA160, and miRNA169, have been identified as being significantly affected during viral infections, correlating with TMV and SMV disease symptoms in plants such as *Nicotiana tabacum* and *Arabidopsis* (Bazzini et al., 2009). In particular, the expression of these miRNAs can be altered by viral proteins that suppress RNA silencing, thereby affecting the ability of plants to mount an effective defense. For example, TuMV interferes with the activity of *miR171*, which plays a role in regulating plant resistance to stress (Kasschau et al., 2003). Research has demonstrated that during infections, such as those caused by the *Rice black-streaked dwarf virus* (RBSDV), numerous miRNAs (such as *miRNa14* and *miRNA16*) exhibit significant alterations in expression levels, indicating their role in the plant response to viral stress (Sun et al., 2015). Furthermore, miRNAs not only target viral genomes directly but also regulate the expression of host genes involved in defense responses. For example, *miR395* and *miR399* have been identified as being responsive to abiotic stresses and SMV viral infections, suggesting their dual roles in plant stress responses (Zhou et al., 2016). Interaction between miRNAs and their target genes is crucial for maintaining a balance between plant growth and defense mechanisms. This intricate relationship underscores the potential of utilizing miRNA pathways to develop resistant crop varieties through biotechnological approaches aimed at enhancing plant antiviral defense. In summary, although considerable advancements have been achieved in understanding miRNAs functions and origins in organelles, further investigation is needed to elucidate the specific pathways and regulatory mechanisms involved in chloroplast and mitochondrial miRNA biogenesis and their roles during viral infections in plants.

### 4.5 Selective mitochondrial/chloroplast-related genes in plant-virus interactions

In this study, we focused on mitochondrial/chloroplast-related hub genes, particularly *CML, POR,* and *CHIL* groups, in plant-virus interactions, while assessing their biological importance in plant species such as *Arabidopsis*, tobacco, and rice.

#### 4.5.1 Calmodulin-like protein

When plants detect molecular patterns of viruses and damaged host cells, they trigger a range of cellular defenses by activating the cell surface recognition receptors found in the plasma membrane (DeFalco and Zipfel, 2021; Köster et al., 2022). Calcium serves as a crucial signaling molecule in plants, interacting with Ca^2+^-binding proteins and channels to facilitate responses to pathogen infection (Jacob et al., 2021). The calcium signaling network in plants comprises various components, including Ca^2+^ channels, Ca^2+^-ATPases, and calcium-dependent proteins, which work together with receptors and TFs to amplify Ca^2+^ signals (Dong et al., 2022; Liu et al., 2022). The calmodulin-like protein (*CML*) family is the main EF-hand Ca^2+^ sensor in plants and is involved in physiological processes associated with calcium signaling (Liu et al., 2022; Xiang et al., 2023; Zeng et al., 2023; Zhang et al., 2022). *CML* proteins play a critical role in plant immune mechanisms against biotic stress, particularly viral pathogens. For example, TMV infection is associated with cytoplasmic *CML30* and can regulate its expression at nucleic acid and protein levels. Silencing *CML* enhances TuMV, TGMV, and TMV infections, whereas its overexpression enhances host-based resistance by activating a Ca^2+^-dependent oxidative stress response (Kamal et al., 2024; Liu et al., 2022; Wang et al., 2023). In particular, this resistance was linked to the regulation of *IP-L*, as co-silencing of *CML* and *IP-L* negated the facilitation of TMV infection caused by *CML* silencing. In addition, signaling network-mediated Ca^2+^/CML is responsible for physiological responses, including cell development, regulation of ROS levels, growth inhibition, and cell death (Liu et al., 2022). These findings provide a theoretical basis that will enable us to develop *A. thaliana*, rice, Tomato, and tobacco with enhanced resistance to TMV in the future.

#### 4.5.2 Protochlorophyllide oxidoreductase

Protochlorophyllide oxidoreductase (*POR*) is a nuclear-encoded protein located in the inner membranes of plastids and is primarily involved in the biosynthesis of chlorophyll. The role of *POR* in chloroplast function and its interaction with viral infections is a critical area of research, particularly for understanding how plants manage oxidative stress and developmental processes under pathogen attack. Research indicates that *Barley stripe mosaic virus* (BSMV) and CMV lead to changes in the lipid composition of etiolate membranes, which affect the translocation and functionality of *POR*, ultimately impairing chloroplast development. This disruption can manifest as chlorotic symptoms in infected plants, owing to the inhibition of pigment synthesis. Specifically, BSMV infection reduces the amount of *POR* present in the etioplasts of infected barley leaves compared to non-infected controls (Bhattacharyya and Chakraborty, 2018; Schemenewitz et al., 2007). This decrease in *POR* disrupts the structure of prolamellar bodies (PLBs), leading to inhibited pigment synthesis and greening processes in infected cells, which manifest as chlorotic stripes on the leaves. The altered expression and localization of *POR* during viral infection may hinder the ability of plants to manage oxidative stress. For example, overexpression of *PORC* has been proposed as a potential biotechnological strategy to enhance crop tolerance to oxidative stress induced by ROS such as singlet oxygen (1O2) (Pattanayak and Tripathy, 2011). Evidence suggests that *POR* is a pivotal component of chloroplast function and development, particularly under stress conditions induced by viral infections. Thus, further exploration of *POR* function could provide valuable insights into plant-virus interactions and potential resistance mechanisms.

#### 4.5.3 Chelatase subunit I

Recent studies have highlighted that viral infections can significantly affect the chloroplast function and chlorophyll biosynthesis. The resulting downregulation of chloroplast and photosynthesis-related genes (CPRGs) is a common feature observed during viral infections (Islam et al., 2020). CMV Y satellite RNA (Y-sat) is a non-coding subviral RNA that can disturb chloroplast function and induce yellowing symptoms in disease tobacco (Jiang and Zhou, 2023; Luo et al., 2013; Shimura et al., 2011; Smith et al., 2011). Chelatase subunit I (*CHLI*) serves as a key regulatory point in chlorophyll biosynthesis by catalyzing the insertion of magnesium into protoporphyrin IX, a critical step in the formation of chlorophyll. Disruption of *CHLI* expression during chloroplast development can lead to the accumulation of chlorophyll intermediates, which may produce ROS, oxidative stress, chlorosis, and cause cellular damage (Islam et al., 2020). Induction of viral symptoms is primarily attributed to siRNA-mediated silencing of magnesium protoporphyrin *CHLI* protein. The *CHLI* mRNA contains a 22-nt complementary sequence derived from the CMV-Y-sat genome, leading to its downregulation in infected plants. Therefore, transgenic plants expressing a silencing-resistant variant of *CHLI* exhibited no symptoms upon CMV-Y-sat infection, reinforcing the notion that *CHLI* silencing is directly responsible for the observed yellowing symptoms in *Arabidopsis* and tobacco (Jiang and Zhou, 2023; Luo et al., 2013; Shimura et al., 2011; Smith et al., 2011). Interestingly, species-specific in symptom development has been linked to natural sequence variations within the *CHLI* gene. For instance, certain tobacco species that do not exhibit yellowing upon Y-Sat infection possess single-nucleotide polymorphisms in the siRNA-targeted region of the *CHLI* transcript. This suggests that variations in the *CHLI* sequence can prevent siRNA-mediated silencing, thereby explaining why some species are resistant to CMV-Y-sat-induced symptoms (Liu et al., 2014; Scholthof, 2011).

In summary, these findings indicate that the silencing of key chloroplast proteins not only affects chlorophyll biosynthesis and its regulation through siRNA, but also activates plant defense mechanisms during viral infections. The ability of CMV Y-Sat to specifically target and downregulate *CHLI* highlights the complex interaction between viral pathogens and host plant gene expression. This research not only elucidates the molecular basis for viral symptomatology but also opens avenues for exploring genetic resistance strategies against viral infections in crop plants. Future research should focus on further elucidating these interactions and exploring potential applications for enhancing resistance against viral infections through genetic modifications targeting key regulatory genes, such as *CHLI*, *POR*, and *CML*.

## 5 Conclusion

Investigation of chloroplast/mitochondria-related gene responses during viral infections is essential for understanding plant defense mechanisms against pathogens. Our findings illustrate that the strategic targeting of these organelles by viruses is not merely a passive consequence of infection but also an active manipulation that facilitates viral replication while simultaneously disrupting host cellular functions. The identification of chloroplast/mitochondria-related DEGs revealed a complex network of defensive strategies employed by plants, such as *Arabidopsis*, tobacco, and rice. Furthermore, comparative analyses across species have provided a foundation for understanding the evolutionary dynamics of plant responses to viruses. The common DEGs highlighted the conserved nature of these interactions across species. The discovery of TFs emphasizes the regulatory layers involved in immune response, suggesting that these factors could serve as potential targets for enhancing resistance against viral pathogens. This study also highlights the importance of miRNAs in mediating defense responses, indicating that post-transcriptional regulation is a critical component of how plants adapt to viral challenges. The presence of novel hub genes with unknown functions presents exciting opportunities for future research aimed at elucidating their roles in plant immunity. Understanding these molecular pathways is crucial for developing genetic engineering strategies for creating virus-resistant crops. Future breeding programs can leverage the identified candidate genes to enhance crop resilience against economically significant viral threats. While progress has been made in identifying key genes involved in plant-virus interactions, substantial gaps remain regarding mitochondrial-specific mechanisms. Future investigations should focus on elucidating the specific interactions between viral proteins and organelle functions to develop targeted strategies for sustainable agriculture in the face of the increasing viral challenges.

## Data Availability

The datasets presented in this study can be found in online repositories. The names of the repository/repositories and accession number(s) can be found in the article/Supplementary material.

## Footnotes

^1^ www.ebi.ac.uk/arrayexpress

^2^ www.ncbi.nlm.nih.gov/geo/

^3^ http://bioinfo.bti.cornell.edu/cgi-bin/itak/index.cgi

^4^ http://plants.ensembl.org

^5^ meme.nbcr.net/meme/intro.html

^6^ http://meme-suite.org/tools/tomtom

^7^ https://string-db.org/
