# Supplementary material for: The crucial role of mitochondrial/chloroplast-related genes in viral genome replication and host defense: integrative systems biology analysis in plant-virus interaction
Source: Front Microbiol. 2025 Apr 23;16:1551123. doi: 10.3389/fmicb.2025.1551123 (PMC12055828; doi:10.3389/fmicb.2025.1551123)
Supplement: Supplementary file 8 [file Table_8.docx]

**Table S8-a. The conserved cis-acting elements found in promoter of Arabidopsis DEGs by the MEME analysis.**

| **Significant GO term identified by GOMO** | **Best match in JASPAR and PLACE** | **Width** | **E-value** | **Motif Logo** | **Motif name** |
| --- | --- | --- | --- | --- | --- |
| MF transcription factor activity CC plasma membrane CC nucleus BP transmembrane receptor protein tyrosine kinase signaling pathway BP regulation of transcription, DNA-dependent | MA1267.1 | 29 | 4.2e-069 | 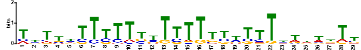 | Motif 1 |
| MF transcription factor activity CC nucleus CC plasma membrane BP regulation of transcription, DNA-dependent MF protein serine/threonine kinase activity | MA1403.1  MA1156.1 | 21 | 2.9e-041 | 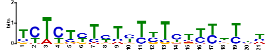 | Motif 2 |
| MF transcription factor activity CC plasma membrane | MA1156.1 | 20 | 1.4e-029 | 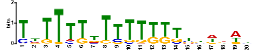 | Motif 3 |
| MF transcription factor activity MF protein serine/threonine kinase activity BP transmembrane receptor protein tyrosine kinase signaling pathway CC nucleus CC chloroplast | MA1723.1  MA1267.1 | 5 | 2.9e-013 | 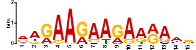 | Motif 4 |
| CC mitochondrion CC chloroplast thylakoid membrane BP mitochondrial transport | \| MA1257.1 \| \| --- \| \|  \| | 29 | 1.3e-006 | 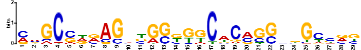 | Motif 5 |
| MF transcription factor activity CC nucleus BP protein amino acid phosphorylation CC cytoplasm MF protein serine/threonine kinase activity | MA1403.1  MA1267.1 | 50 | 1.1e-003 | 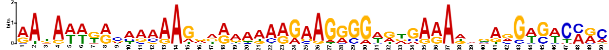 | Motif 6 |
|  | MA1277.14  MA1403.1 | 40 | 2.6e-001 | 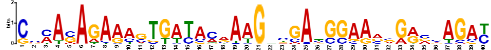 | Motif 7 |
| MF transcription factor activity CC plasma membrane BP regulation of transcription, DNA-dependent | MA1868.1 | 15 | 6.4e-003 | 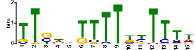 | Motif 8 |
| MF structural constituent of ribosome BP translation CC mitochondrion CC chloroplast envelope CC ribosome | MA1863.1 | 50 | 7.8e-001 | 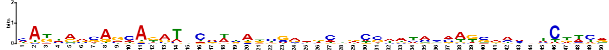 | Motif 9 |
| CC nucleus MF transcription factor activity CC chloroplast envelope CC chloroplast stroma MF protein binding | MA1267.1 | 41 | 7.1e-001 | 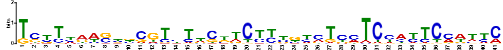 | Motif 10 |
| MF transcription factor activity BP regulation of transcription | MA1268.1  MA1214.1 | 28 | 1.6e+001 | 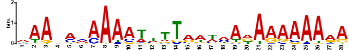 | Motif 11 |

**Table S8-b. The conserved cis-acting elements found in promoter of Tobacco DEGs by the MEME analysis.**

| **Significant GO term identified by GOMO** | **Best match in JASPAR and PLACE** | **Width** | **E-value** | **Motif Logo** | **Motif name** |
| --- | --- | --- | --- | --- | --- |
| MF transcription factor activity CC nucleus CC plasma membrane BP transmembrane receptor protein tyrosine kinase signaling pathway MF protein serine/threonine kinase activity | MA0078.2 | 21 | 4.0e-074 | 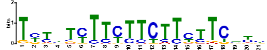 | Motif 1 |
| MF transcription factor activity CC plasma membrane CC nucleus | MA0143.4  MA0277.1 | 29 | 3.9e-054 | 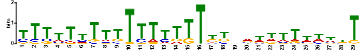 | Motif 2 |
| - | MA0522.3 | 41 | 8.1e-045 | 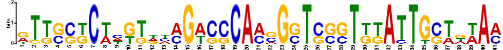 | Motif 3 |
| MF transcription factor activity CC nucleus CC plasma membrane MF protein binding BP protein amino acid phosphorylation | MA0554.1 | 15 | 5.5e-029 | 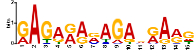 | Motif 4 |
| - | MA0558.1 | 50 | 1.8e-026 | 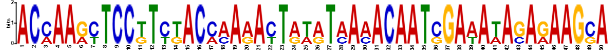 | Motif 5 |
| - | MA0687.1 | 41 | 8.4e-026 | 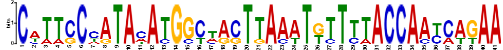 | Motif 6 |
| - | MA0868.2  MA0909.3 | 50 | 2.4e-026 | 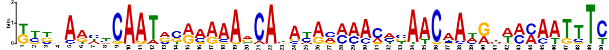 | Motif 7 |
| CC nucleus MF transcription factor activity CC plasma membrane MF protein binding BP protein amino acid phosphorylation | MA1012.1 | 41 | 5.7e-031 | 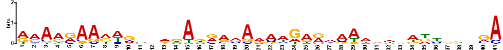 | Motif 8 |
| - | MA1157.1 | 50 | 2.7e-026 | 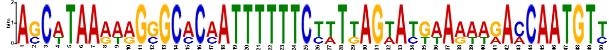 | Motif 9 |
| - | MA1159.1 | 50 | 6.4e-022 | 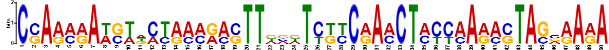 | Motif 10 |
| MF transcription factor activity | MA1204.1  MA1184.1 | 50 | 2.2e-021 | 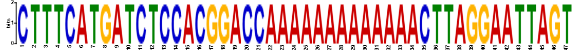 | Motif 11 |

**Table S8-c. The conserved cis-acting elements found in promoter of Rice DEGs by the MEME analysis.**

| **Significant GO term identified by GOMO** | **Best match in JASPAR and PLACE** | **Width** | **E-value** | **Motif Logo** | **Motif name** |
| --- | --- | --- | --- | --- | --- |
| MF transcription factor activity CC plasma membrane CC nucleus BP regulation of transcription, DNA-dependent BP transmembrane receptor protein tyrosine kinase signaling pathway | MA1875.1 MA1268.1 | 20 | 6.3e-066 | 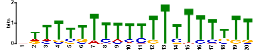 | Motif 1 |
| MF transcription factor activity CC nucleus CC plasma membrane MF protein binding BP protein amino acid phosphorylation | MA1267.1  MA1403.1 | 38 | 4.6e-055 | 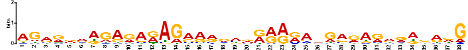 | Motif 2 |
| MF transcription factor activity CC nucleus CC chloroplast CC plasma membrane BP regulation of transcription, DNA-dependent | [MA1267.1](http://jaspar2022.genereg.net/matrix/MA0080.6) | 21 | 6.6e-023 | 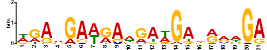 | Motif 3 |
| CC chloroplast thylakoid membrane MF ATP binding MF transcription factor activity | [MA1262.1](http://jaspar2022.genereg.net/matrix/MA0456.1) | 48 | 5.5e-020 | 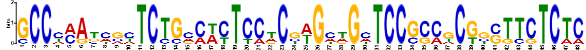 | Motif 4 |
| MF transcription factor activity CC nucleus MF ATP binding CC chloroplast stroma CC chloroplast thylakoid membrane | MA1714.1 | 49 | 1.6e-023 | 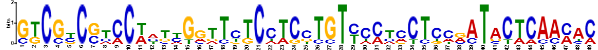 | Motif 5 |
| CC chloroplast | MA1261.1 | 49 | 8.5e-019 | 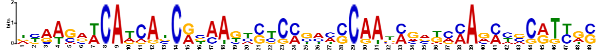 | Motif 6 |
| - | \| MA1119.1 \| \| --- \| \| MA1419.1 \| | 50 | 5.1e-018 | 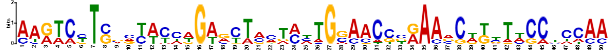 | Motif 7 |
| CC chloroplast MF transcription factor activity | MA1267.1  MA1403.1 | 50 | 1.1e-018 | 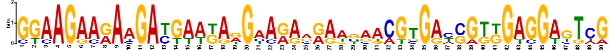 | Motif 8 |
| MF transcription factor activity CC plasma membrane BP transmembrane receptor protein tyrosine kinase signaling pathway CC nucleus BP protein amino acid phosphorylation | MA1875.1 | 15 | 3.4e-015 | 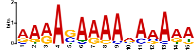 | Motif 9 |
| - | MA0518.1 | 41 | 6.3e-014 | 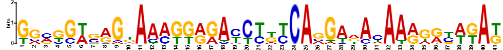 | Motif 10 |
| MF transcription factor activity CC plasma membrane CC nucleus BP flower development BP regulation of transcription, DNA-dependent | MA1267.1  MA1268.1 | 29 | 1.1e-012 | 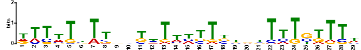 | Motif 11 |
