# Supplementary material for: The crucial role of mitochondrial/chloroplast-related genes in viral genome replication and host defense: integrative systems biology analysis in plant-virus interaction
Source: Front Microbiol. 2025 Apr 23;16:1551123. doi: 10.3389/fmicb.2025.1551123 (PMC12055828; doi:10.3389/fmicb.2025.1551123)
Supplement: Supplementary file 10 [file Data_Sheet_1.docx]

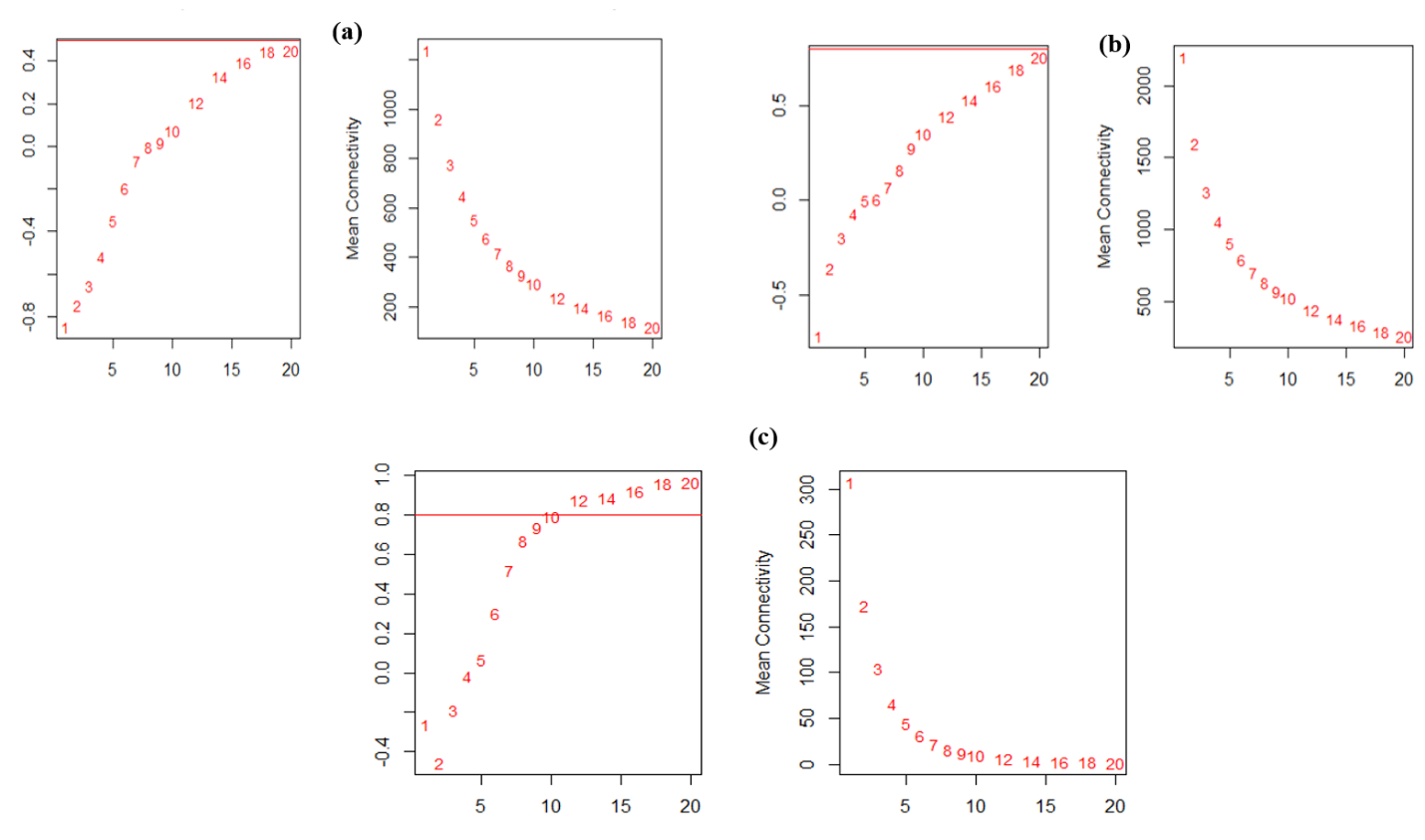


Supplementary Figure 1. Determination of soft-thresholding power (β) in the co-expression network analysis and module identification of (a) Arabidopsis, (b) tobacco and (c) rice plants challenged by virus. The left panel shows the analysis of the scale-free fit index for various soft-thresholding powers (β). The right panel displays the analysis of the mean connectivity (degree, y-axis) for various soft-thresholding (x-axis) powers. Red dots show the up-regulated genes. The soft-thresholding power can be attained at the scale-free model fitting index R2 < 0.8.
