# Supplementary material for: The crucial role of mitochondrial/chloroplast-related genes in viral genome replication and host defense: integrative systems biology analysis in plant-virus interaction
Source: Front Microbiol. 2025 Apr 23;16:1551123. doi: 10.3389/fmicb.2025.1551123 (PMC12055828; doi:10.3389/fmicb.2025.1551123)
Supplement: Supplementary file 11 [file Data_Sheet_2.docx]

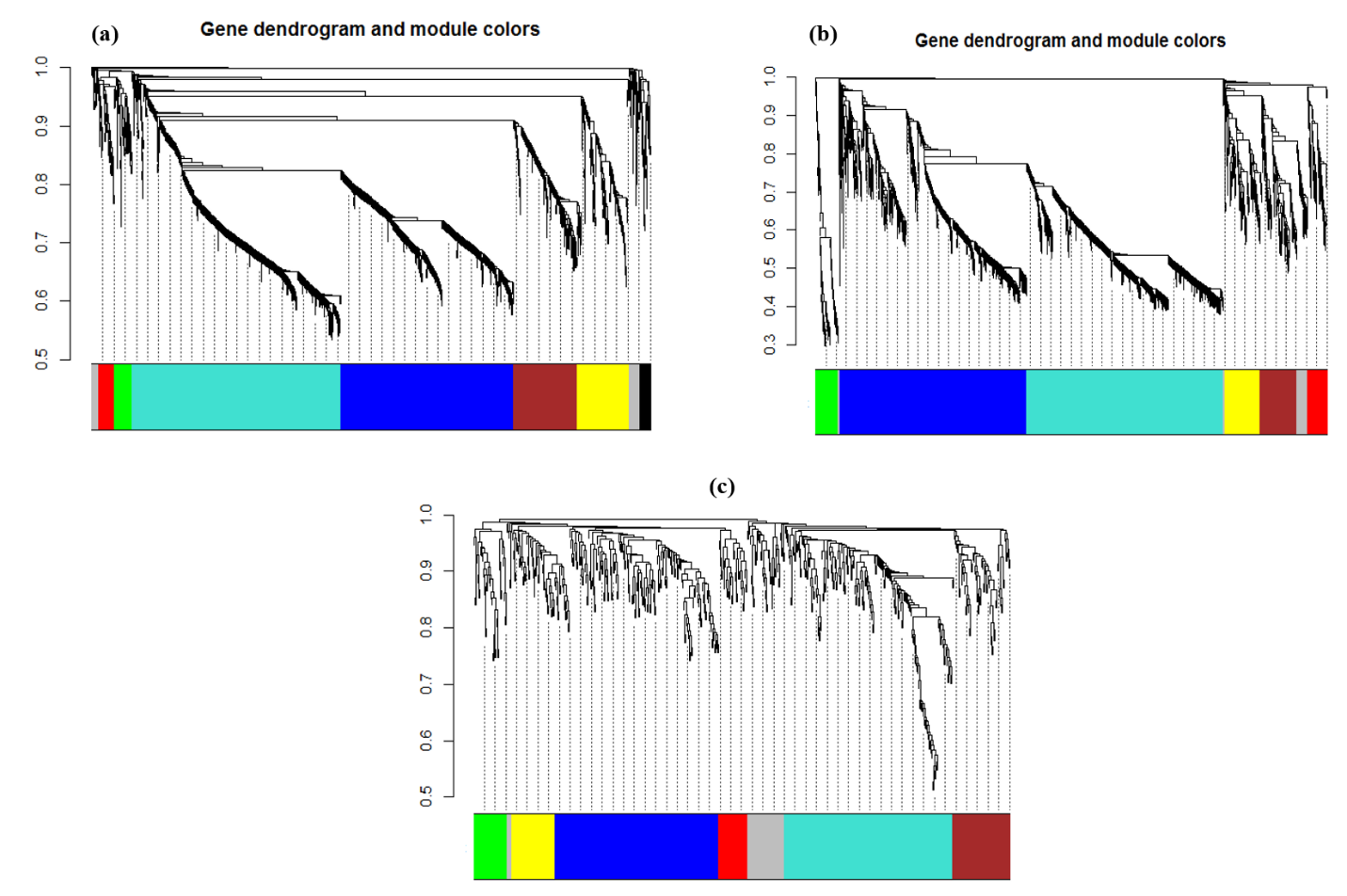


Supplementary Figure 2. Hierarchical clustering tree for co-expression modules identified by weighted gene co-expression network analysis (WGCNA). The color labels correspond to the different co-expression modules identified in differentially expressed genes of (a) Arabidopsis, (b) tobacco and (c) rice plants in response to viral infection.
