# Supplementary material for: The crucial role of mitochondrial/chloroplast-related genes in viral genome replication and host defense: integrative systems biology analysis in plant-virus interaction
Source: Front Microbiol. 2025 Apr 23;16:1551123. doi: 10.3389/fmicb.2025.1551123 (PMC12055828; doi:10.3389/fmicb.2025.1551123)
Supplement: Supplementary file 12 [file Data_Sheet_3.docx]

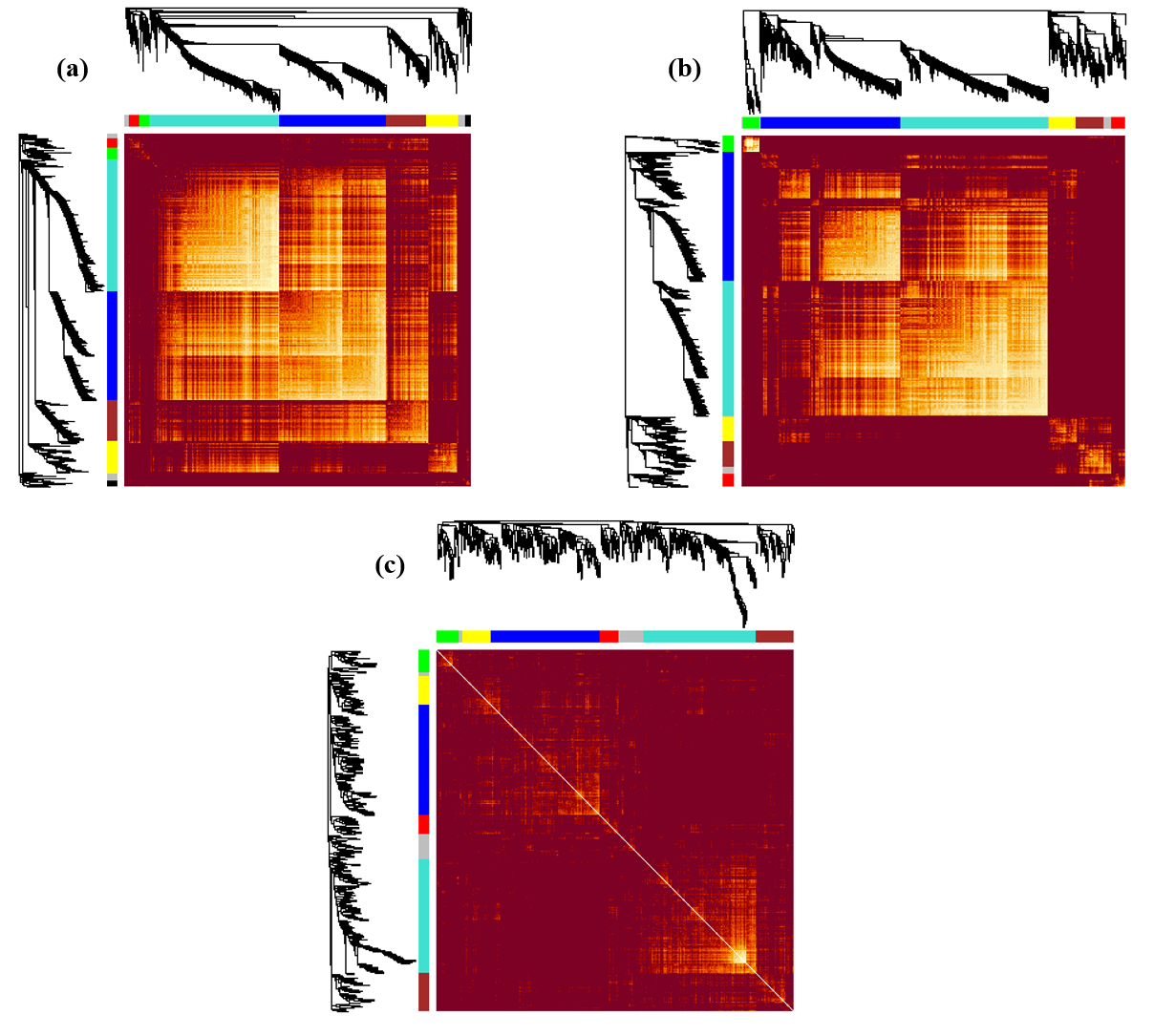


Supplementary Figure 3. Heatmap plot of topological overlap in the co-expression network modules of *Arabidopsis* (a), tobacco (b) and rice (c) DEGs during the viral infection. In the heatmap, each row and column correspond to a gene, light color denotes high topological overlap, and progressively darker red denotes low topological overlap. In the TOM plot, darker red color shows low overlap as progressively light color represents higher overlap among DEGs. Blocks of darker colors along the diagonal correspond to the modules. Gene dendrograms and module assignments are also presented on the left side and the top.
